# A Fundamental Difference in the Nature of Personal Values and Personality Traits Revealed Through Different Patterns of Stability Across Their Distributions

**DOI:** 10.1111/jopy.12979

**Published:** 2024-10-04

**Authors:** Joshua Lake, Anat Bardi, Joanne Sneddon, Julie A. Lee

**Affiliations:** ^1^ Centre for Human and Cultural Values and UWA Business School University of Western Australia Perth Western Australia Australia; ^2^ Department of Psychology Royal Holloway University of London Egham UK

**Keywords:** change, personal values, personality traits, quantile correlations, stability

## Abstract

**Objective:**

Personal values and personality traits are both important aspects of personality, but much is still unknown about the fundamental differences between the constructs, including how their patterns of temporal stability compare. This paper investigated patterns of intra‐individual stability in both values and traits.

**Method:**

Quantile correlations were estimated between each of the 20 refined personal values and the same values 2 years later in a large longitudinal sample of Australian adults (*N* = 2875). The same was done for each of the 15 Five‐Factor Model trait facets in a subsample of these participants (*n* = 2424).

**Results:**

It was observed that more important values tended to remain more stable over time, while traits retained a similar stability regardless of trait strength, and frequently showed small decreases in stability at extreme levels.

**Conclusions:**

Findings indicate that highly prioritized values may be a more central aspect of the self, and a more reliable element for predicting future outcomes, than less highly prioritized values, but in contrast, traits do not function in a way that is dependent on trait strength.

## 1 Introduction

Personal values are understood to be an important aspect of personality (see e.g., Baumert et al. 2017). They are systematically related to other aspects of personality, such as traits (see meta‐analyses in Fischer and Boer 2015; Parks‐Leduc, Feldman, and Bardi 2015), but they differ conceptually to traits, and they also differ in how closely they are associated with different kinds of outcomes (Pozzebon and Ashton 2009; Roccas et al. 2002; Skimina and Cieciuch 2020). Much is still unknown about the nature and extent of differences between these two constructs. One lens through which the differences between personal values and traits may be revealed is by examining within‐person patterns of stability over time. If it is the case that these patterns differ meaningfully and systematically between values and traits, this might shed more light on the fundamental differences between these two aspects of personality. In this paper, patterns of intra‐individual change in personal values and in personality traits over 2 years in a large varied longitudinal sample are examined.

### 1.1 Personal Values and Personality Traits

#### 1.1.1 Similarities and Differences

Personal values and personality traits are both important aspects of personality, which share key similarities. They are both rather stable elements of personality within adults over time (e.g., Dobewall and Aavik 2016). They also share similar levels of heritability (e.g., Bouchard Jr and McGue 2003; Twito and Knafo‐Noam 2020) and common genetic factors (Schermer et al. 2011), and are both associated with some similar outcomes, such as behaviors (e.g., Skimina, Cieciuch, and Strus 2021) and political ideology (e.g., Costello et al. 2022).

Values and traits also sometimes share common content. For instance, kind people (i.e., with high levels of this trait) are likely to also see kindness as being important as a broad motivational goal (i.e., to place a high importance on these values). When there is content overlap between values and traits, they have been found to be systematically related (see meta‐analyses in Fischer and Boer 2015; Parks‐Leduc, Feldman, and Bardi 2015). Some researchers argue that the two constructs are not truly distinct, with values being seen as surface manifestations of basic traits (e.g., McCrae and Costa Jr. 2008). There is some evidence for this, in that traits predict later values more strongly than values predict later traits (e.g., Fetvadjiev and He 2019; Vecchione et al. 2019).

However, personal values and personality traits differ conceptually. Personal values are expressive of broad, abstract motivational goals that people aspire to (e.g., Schwartz 1992), whereas personality traits are commonly defined as representing consistent, enduring dispositions (Deary 2009; McCrae 2009). That is, values are defined as being internal motivations (Schwartz 1992), whereas traits more generally describe characteristic tendencies (see John and Srivastava 1999). While they can refer to the same content (e.g., kindness), their most basic difference is whether this content references an individual's motivations (i.e., value kindness; being kind is an important motivator to them), or their patterns of behavior, thought, and affect more generally (i.e., trait kindness; they tend to exhibit kindness).

There are also differences in how closely the two constructs are associated with different outcomes. For instance, values have been found to relate to religiosity (Roccas et al. 2002) and voting behavior (Caprara et al. 2006) more strongly than traits. In contrast, traits relate to emotional outcomes, such as well‐being, more strongly than values (Fetvadjiev and He 2019; Roccas et al. 2002). Some researchers argue that values and traits are not only distinct conceptually, but that they both uniquely contribute to the understanding of personality and are both uniquely influenced by genetic and environmental factors (e.g., neo‐socioanalytic theory; Roberts and Nickel 2017). Ultimately, we are still far from truly understanding the degree to which traits and values are distinct aspects of personality. One way of trying to gain further insight may be through an examination of their patterns of stability and change.

#### 1.1.2 Stability and Change

Current research suggests that values and traits have similar stability (e.g., Dobewall and Aavik 2016). Both values and traits develop throughout the life span (e.g., Damian et al. 2019; Vecchione et al. 2020), becoming increasingly stable throughout adolescence and then remaining relatively stable in adulthood (e.g., Benish‐Weisman, Daniel, and Knafo‐Noam 2017; Bleidorn et al. 2022; Hopwood et al. 2011). They also tend to be vulnerable to change in response to major life events that require adjustment in multiple aspects of a person's life (e.g., Bardi et al. 2014; Löckenhoff et al. 2009). But this is not to say that values and traits necessarily operate in the same way with regard to their stability. We argue that value stability is likely to be dependent on value importance, but trait stability is less likely to be dependent on trait strength.

According to knowledge on cognitive schemas, repeated value‐expressive behavior has the potential to act reciprocally and strengthen a value (see Bardi and Goodwin 2011). Recent studies have shown that most highly important values are more strongly associated with behavior than those that are less important, showing systematic patterns of non‐linearity (Lake et al. 2024; Lee et al. 2022). For instance, Lake et al. (2024) found that traditional values were highly correlated with their behavioral index, one (*r* = 0.63) and 2 years later (*r* = 0.66), at the 90th percentile of value importance and much more weakly correlated, one (*r* = 0.22) and 2 years later (*r* = 0.20), at the 10th percentile of value importance, based on quantile correlations. Given the reciprocal nature of value‐behavior relations and evidence that highly important values are more strongly associated with behavior, we might also expect that highly important values will be more stable over time.

By contrast, there is no suggestion in the literature that trait stability should differ across the distribution of trait strength, and no evidence that relations between traits and behaviors are non‐linear. Thus, we might expect that a small increase in a trait should be accompanied by a small increase in associated behaviors, no matter how strongly characteristic the trait is. This may be in part because traits are descriptive of patterns of behavior. However, it may also be a function of the independent nature of traits, where a person's standing on one trait (e.g., conscientiousness) does not inform their standing on another (e.g., open‐mindedness). In contrast to values, which are theoretically structured on a circumplex of underlying compatible and conflicting motivations (Schwartz 2012), traits are most often derived from a factor solution designed to elicit maximally independent characteristics (Soto and John 2017). On this basis, we have no reason to expect that trait stability should differ across the distribution of trait strength.

### 1.2 The Current Study

In order to examine whether values and traits exhibit different characteristic patterns of stability, we examined value and trait stability in two complementary analyses. In Part 1, quantile models of the correlation between each of the 20 refined personal values at T1, and the same value at T2 (2 years later) were constructed. In Part 2, the same was done for each of the 15 Five‐Factor Model trait facets.

It is hoped that by investigating the characteristic patterns of stability in personal values and personality traits, and whether they relate to value importance and trait strength, respectively, we will be able to further our understanding of the relations, and distinctions, between values and traits, and how they function together as complementary constituent elements of personality.

## 2 Materials and Methods

The current study was part of a larger longitudinal project drawing on the Pureprofile consumer panel, which was fielded in three waves in 2017, 2018, and 2019. In each wave, participants completed a series of short (5–10 min) online survey modules over several weeks. This was done in order to reduce respondent fatigue and common method bias (Hulland, Baumgartner, and Smith 2018). The first survey measured respondents' personal values and the second their personality traits. All survey modules can be viewed by following this link: https://osf.io/w6uen/, and data collection was approved by The University of Western Australia Human Research Ethics Committee (RA/4/1/8647).

### 2.1 Participants and Procedure

Part 1 included all respondents (*N* = 2875) who completed the values survey during both the first (T1, 2017) and third (T2, 2019) waves.^1^ Mean age at T1 was 53.9 years (SD = 14.0; range 18–75 years), with 60% being women. Almost all respondents self‐reported as Australian citizens (91%) or residents (8%). The highest level of completed education was elicited at T2, with 32% reporting completing some or all high school, 35% reporting a technical or trade certificate, 23% an undergraduate degree, and 10% a postgraduate degree.

Part 2 included all respondents (*N* = 2424) who completed the traits survey during both the first (T1, 2017) and third (T2, 2019) waves. Mean age at T1 was 53.0 years (SD = 14.0), with a minimum of 18 and maximum of 75, and 62% of respondents were women. Almost all respondents self‐reported as Australian citizens (92%) or residents (8%). Education levels were the same as in Part 1.

#### 2.1.1 Attrition Analyses

There was significant attrition in participant numbers between T1 (2017) and T2 (2019); however, most of the attrition was in the first year. This may be due to panel churn, as almost all of those who remained in the study across the first two waves, continued to the third wave. Specifically, of the 7453 respondents who completed the values measured in the first wave, 45% completed it in the second wave, and of these, 86% also completed it in the third wave. Of the 6644 respondents who completed the trait measure in the first wave, 40% completed it in the second wave, and of these, 92% also completed it in the third wave. To mitigate attrition as much as possible, recontact reminders were sent to participants (at least four times), and surveys were kept open for several weeks during this time.

To examine potential bias due to attrition, respondents who completed both the first wave (T1, 2017) and the third wave (T2, 2019) were compared with those who failed to complete the third wave. A MANOVA on the 20 refined values revealed significant differences (*F*_(21, 7431)_ = 39.47, *p* < 0.001), as did a MANOVA on the 15 trait facets (*F*_(16, 6630)_ = 36.19, *p* < 0.001). Those who completed surveys over the 2‐year period were older (*F*_(1,7451)_ = 757.21, *p* < 0.001). They were also higher on self‐direction (thought), security (personal and societal), conformity (rules), and benevolence (dependability and caring), and lower on stimulation, hedonism, achievement, power (resources and dominance), and face (ranging from achievement values [*F*_(1,7451)_ = 225.72, *p* < 0.001] to self‐direction (thought) values [*F*_(1,7451)_ = 6.37, *p* = 0.01]; see Appendix A, Table S3). They were also higher on agreeableness (compassion, respectfulness, and trust), conscientiousness (organization, productiveness, and responsibility) and open‐mindedness (creative imagination) and lower on negative emotionality (anxiety, depression, and emotional volatility) (ranging from conscientiousness (responsibility) [*F*_(1,6645)_ = 130.03, *p* < 0.001], to open‐mindedness (creative imagination) [*F*_(1,6645)_ = 5.87, *p* = 0.02]; see Appendix A, Table S4).

### 2.2 Measures

#### 2.2.1 Personal Values

Personal values were measured with the Schwartz Best Worst Values (Refined) survey (BWVr; Lee et al. 2019). In this survey, respondents are asked to choose the value item that is most important and the value item that is least important from subsets of 5 value items. In total, 21 value subsets are presented, based on a Youden balanced incomplete block experimental design, where each value item is seen 5 times, and each pair of value items is seen together once across all subsets. Because values are generally viewed as desirable, respondents tend to rate most value items in rating scales from “somewhat important” to “very important” (Schwartz and Bardi 2001). Given this, Schwartz (1992) suggested ipsatizing value rating scale scores, by taking the mean of all value items from each item score to account for this response bias and produce a measure of relative value importance. In this paper, value priorities were elicited directly using the BWVr, which measures relative value importance in a series of forced‐choice comparisons (Lee et al. 2019). Importantly, the BWVr can be scored to produce a more fine‐grained scale, with greater variance across the distribution of value scores, than values rating scales.

Prior to scoring the value items, reliability was assessed by examining the consistency of choices. Specifically, the proportion of times each value item was chosen as most important by each respondent across all subsets was examined. Consistency was high, with 92% of respondents choosing at least one value item as most important four or five of the five times it appeared and 7% three of the five times it appeared (see Appendix C, Table S6). Respondent scores were obtained for each of the 20 value items using the square root ratio of most‐to‐least counts (see Lee, Soutar, and Louviere 2008 for a detailed description of the scoring procedure and formula). This method is recommended as it produces more scale points (i.e., 20) than other best‐worst scoring procedures (see Lake et al. 2024; Lee et al. 2022). Scores using this method range from 0.25 to 4.00. See Appendix A, Table S1 for descriptive statistics.

#### 2.2.2 Personality Traits

Personality traits were measured using the Big Five Inventory 2 (BFI‐2) (Soto and John 2017), which measures the “Big 5” traits and three constituent facets of each trait. The BFI‐2 comprises 60 items, 12 relating to each of the 5 traits (4 to each facet), scored on a 5‐point scale (1 = *disagree strongly*, 2 = *disagree a little*, 3 = *neutral/no opinion*, 4 = *agree a little*, 5 = *agree strongly*). Items are content‐balanced, with half in each facet being true‐keyed and half false‐keyed. All items were presented in random order to each respondent. As suggested by Soto and John (2017), acquiescence was controlled for at the item level, by centering each respondent's item responses around their within‐person mean, without reversing the false‐keyed items. Individual respondent's mean scores for each of the 15 facets were then calculated. See Appendix A, Table S2 for descriptive statistics, and Appendix C, Tables S7–S9 for scale reliability analysis.

### 2.3 Analytical Strategy

Quantile correlations (Choi and Shin 2022), which are derived from quantile regression (Koenker and Bassett 1978), were used to assess whether value and trait stability are stronger at higher and weaker at lower levels of value importance and trait strength. Ordinary quantile regression examines the effect of predictor variables on different quantiles (*τ*) of an outcome variable, where *τ ∈* (0, 1).^2^ This method differs from standard linear regression, which minimizes the sums of squared residuals to estimate models for conditional *mean* functions; quantile regression estimates models for conditional *quantile* (e.g., median) functions (see Koenker and Hallock 2001). A quantile (*τ*) simply represents a point of interest along the distribution of an outcome. For instance, the 0.5 quantile (*τ* = 0.5) represents the median or 50th percentile of the distribution, whereas the 0.3 quantile (*τ* = 0.3), represents the 30th percentile of the distribution. Examining quantile functions enables researchers to investigate whether an outcome variable is more closely related to its predictor(s) at some points along its distribution than others. Importantly, quantile regression operates by differentially weighting residuals rather than splitting data into groups: the entire dataset is used in estimations at any given quantile (see Koenker and Hallock 2001).

Choi and Shin (2022) extended the quantile regression method to develop quantile correlations, which examine relations across the joint distribution of two variables, rather than solely across the distribution of an outcome variable. This approach is particularly useful for designs such as the present study, where we wish to consider differences in stability across the distribution of values or traits at T1, rather than only at T2.

A quantile correlation coefficient is defined as the geometric mean of two quantile regression slopes; Y on X and X on Y. Quantile correlations thus represent “overall *τ*‐tail dependence in the sense of sensitivity of the conditional *τ*‐quantile of one variable with respect to changes in the other variable” (Choi and Shin 2022, 1079). This coefficient has similar properties to the Pearson correlation coefficient in that it “equals zero for independent pairs of random variables, it equals ±1 for perfectly linearly related pairs, it features scale‐location equivariance, and it is bounded by 1 in absolute value for the general class of random pairs” (Choi and Shin 2022, 1078). It is also comparable across quantiles and with Pearson correlations, within a given distribution. As such, it presents an effective way to examine whether value or trait stability over 2 years differs along the distribution of value importance and trait strength, respectively. The analysis in Part 1 of the present study examined whether stability in values differed by value importance. First, simple (Pearson) correlations were calculated between each of the 20 refined values at the two timepoints (T1, 2017; T2, 2019). Then the quantile correlation method of Choi and Shin (2022) was used to estimate correlations around specific levels of value importance. Correlations between the T1 and T2 values were estimated around seven quantiles (*τ* = 0.2, 0.3, 0.4 … 0.8, representing the 20th, 30th, 40th … 80th percentiles). These quantiles were chosen to avoid the extreme ends of the distribution of value importance, and thus alleviate the need to “jitter” (i.e., add small perturbations to) the data, as was necessary in Lee et al. (2022), who examined nine quantiles (*τ* = 0.1, 0.2, 0.3 … 0.9). Bootstrapping (1000 resamples) was used to estimate 95% confidence intervals conditioned around each quantile. These were adjusted to be consistent with Zou's (2007) procedure in order to enable comparison between correlation estimates at each quantile (*τ* = 0.2, 0.3, 0.4 … 0.8) with the Pearson correlation and the estimate at the median (*τ* = 0.5), to evaluate significant differences. Part 2 followed the same strategy as Part 1, substituting each of the 15 personality trait facet scores at T1 and T2 for refined values. All data and syntax used to produce these results can be accessed at the following link: https://osf.io/s2c74/?view_only=39039a43acf248ee81b9f6145bad9b60.

The required sample size for quantile correlation depends on the chosen quantiles examined, the effect size, and distribution of the data. To estimate the necessary sample size, the R function, power.rq.test was used (Gong 2016), which assesses adequacy of sample size for quantile regression. In the present analyses, the samples met the sample size requirement for estimating quantile correlations at the *τ* = 0.2, 0.3, 0.4 … 0.8 quantiles with a power of 0.80 and a *p*‐value of 0.05 (see Supporting Information, Appendix B for details).

It is also important to note that quantile regression, upon which quantile correlation is based, was designed for use with continuous outcome variables (Huang et al. 2017). It is for this reason that item scoring using the square root ratio of most‐to‐least counts was adopted in the present study for values; alternative scoring methods produce relatively fewer scale points resulting in insufficiently continuous data for successful quantile regression and quantile correlation modeling.

## 3 Results

**FIGURE 1:**
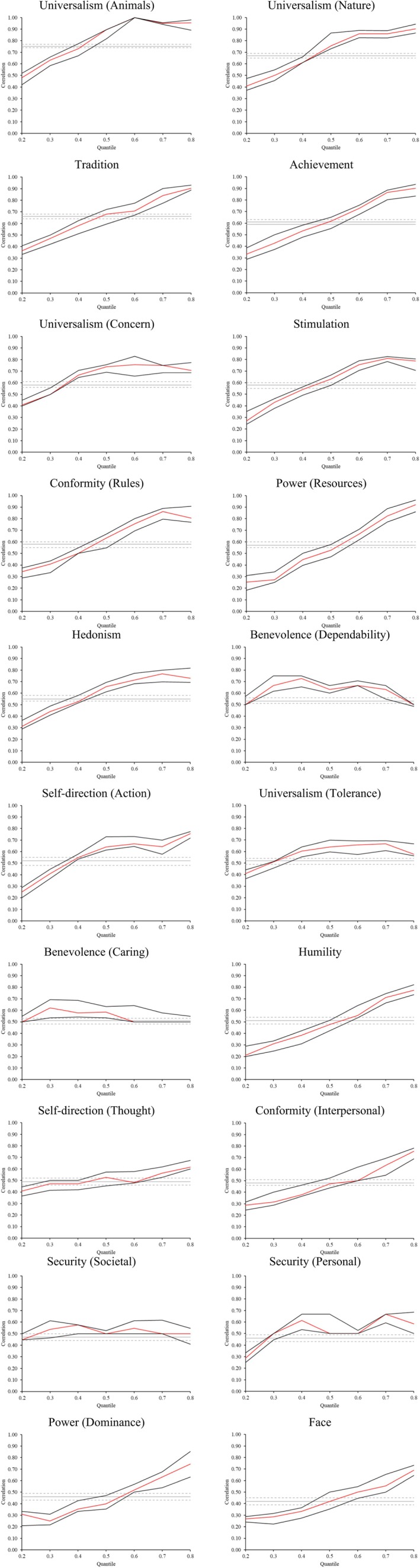
Value stability estimates across the value importance distribution. *n* = 2875. Gray lines represent the Pearson correlation, with dotted lines at 95% confidence intervals; red lines represent quantile correlations, with black lines at 95% confidence intervals. Figures are ordered by the strength of Pearson correlation.

**FIGURE 2:**
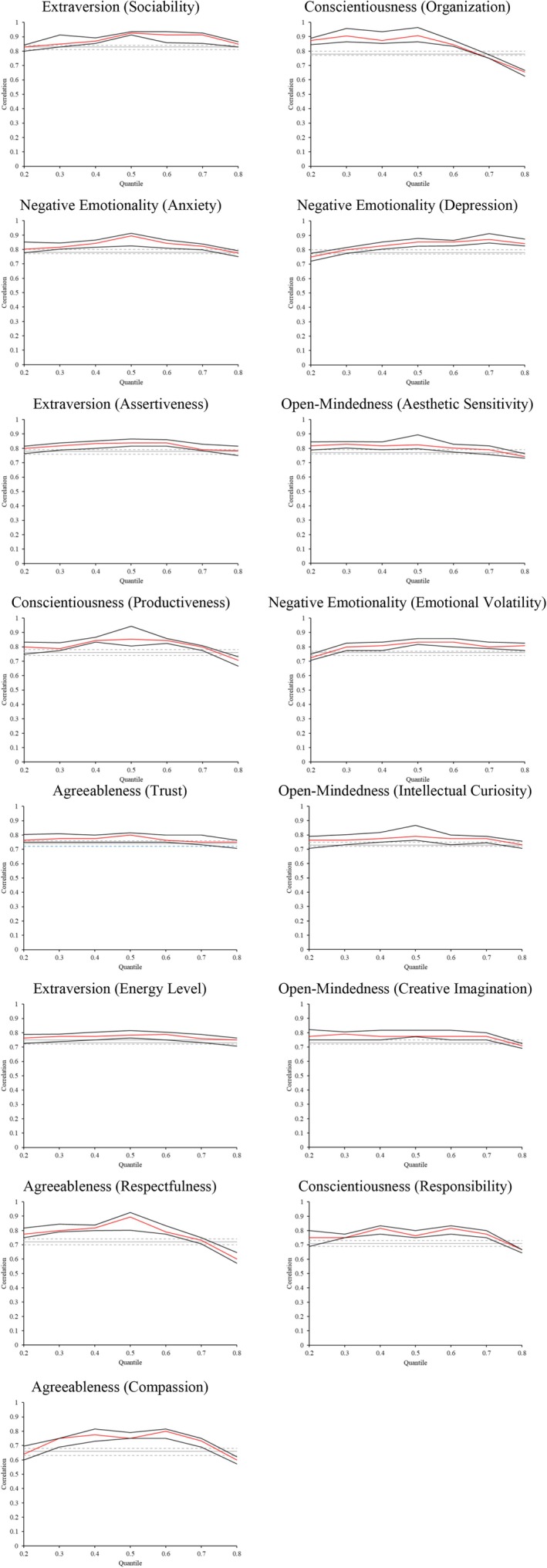
Trait stability estimates across the trait strength distribution. *n* = 2424. Gray lines represent the Pearson correlation, with dotted lines at 95% confidence intervals; red lines represent quantile correlations, with black lines at 95% confidence intervals. Figures are ordered by the strength of Pearson correlation.

Figures 1 and 2 present the results graphically of Pearson and quantile correlations for value and trait stability over 2 years, respectively, together with their 95% confidence intervals. The horizontal axes indicate the quantiles of T1 value importance and T1 trait strength. The solid red line represents the estimated quantile correlations at the 20th, 30th, 40th, 50th, 60th, 70th, and 80th percentile of value importance (i.e., *τ* = 0.2, 0.3, 0.4, 0.5, 0.6, 0.7, 0.8), with the black lines representing their 95% confidence intervals. The gray lines show the Pearson correlations and their confidence intervals (dotted). Each plot in Figure 1 allows the comparison of Pearson correlations with quantile correlations across the entire distribution of value importance at T1; similarly, each plot in Figure 2 allows the comparison of Pearson correlations with quantile correlations across the entire distribution of trait strength at T1.

Tables 1 and 2 provide the Pearson and quantile correlation estimates. Quantile correlations highlighted in green are significantly stronger than those at the median (*τ* = 0.5); quantile correlations highlighted in yellow are significantly weaker than those at the median (95% confidence intervals, bootstrapped with 1000 resamples). For example, in Table 1, for the universalism (animals) value, the quantile correlation estimate at *τ* = 0.2 (0.41) is significantly lower than the estimate at the median (0.76) and is highlighted in yellow accordingly. See Appendix D, Tables S10 and S11 for specific values of confidence interval estimates.

**TABLE 1:** Quantile correlation results: personal values.

Value (refined)	Pearson	*τ* = 0.2	*τ* = 0.3	*τ* = 0.4	*τ* = 0.5	*τ* = 0.6	*τ* = 0.7	*τ* = 0.8
Universalism (animals)	0.75	0.48	0.63	0.73	0.89	1.00	0.95	0.95
Universalism (nature)	0.67	0.41	0.50	0.61	0.76	0.86	0.86	0.90
Tradition	0.66	0.37	0.47	0.58	0.68	0.71	0.84	0.90
Achievement	0.61	0.33	0.43	0.53	0.62	0.73	0.87	0.90
Universalism (concern)	0.58	0.41	0.50	0.67	0.74	0.76	0.75	0.71
Stimulation	0.58	0.27	0.43	0.54	0.63	0.76	0.81	0.79
Conformity (rules)	0.58	0.34	0.41	0.50	0.63	0.76	0.86	0.80
Power (resources)	0.57	0.25	0.27	0.45	0.53	0.67	0.82	0.92
Hedonism	0.55	0.31	0.44	0.53	0.66	0.71	0.77	0.73
Benevolence (dependability)	0.53	0.50	0.67	0.73	0.63	0.67	0.63	0.50
Self‐direction (action)	0.52	0.25	0.41	0.55	0.64	0.67	0.64	0.76
Universalism (tolerance)	0.52	0.41	0.51	0.60	0.64	0.66	0.67	0.58
Benevolence (caring)	0.51	0.50	0.62	0.58	0.59	0.50	0.50	0.50
Humility	0.51	0.21	0.31	0.38	0.48	0.55	0.71	0.77
Self‐direction (thought)	0.49	0.41	0.47	0.47	0.53	0.48	0.56	0.62
Conformity (interpersonal)	0.48	0.29	0.32	0.38	0.47	0.50	0.63	0.76
Security (societal)	0.47	0.45	0.54	0.58	0.50	0.55	0.50	0.50
Security (personal)	0.46	0.29	0.50	0.61	0.50	0.50	0.67	0.58
Power (dominance)	0.46	0.31	0.25	0.35	0.40	0.52	0.63	0.75
Face	0.42	0.27	0.29	0.33	0.42	0.50	0.55	0.69

*Note: n* = 2875. Each row presents the estimated correlations for value stability over a 2‐year period (ordered by Pearson correlation strength). Quantile correlations highlighted in green are significantly stronger than the median (*τ* = 0.5) quantile correlation, and those highlighted in yellow are significantly weaker.

**TABLE 2:** Quantile correlation results: personality traits.

Trait (Facet)	Pearson	*τ* = 0.2	*τ* = 0.3	*τ* = 0.4	*τ* = 0.5	*τ* = 0.6	*τ* = 0.7	*τ* = 0.8
Extraversion (Sociability)	0.83	0.83	0.85	0.87	0.93	0.91	0.91	0.85
Conscientiousness (Organization)	0.78	0.88	0.90	0.88	0.91	0.85	0.75	0.65
Negative Emotionality (Anxiety)	0.78	0.80	0.82	0.84	0.89	0.84	0.82	0.77
Negative Emotionality (Depression)	0.78	0.75	0.80	0.83	0.85	0.85	0.87	0.84
Extraversion (Assertiveness)	0.78	0.80	0.82	0.83	0.84	0.84	0.79	0.78
Open‐Mindedness (Aesthetic Sensitivity)	0.77	0.82	0.83	0.82	0.82	0.80	0.79	0.74
Conscientiousness (Productiveness)	0.76	0.80	0.79	0.85	0.85	0.85	0.80	0.71
Negative Emotionality (Emotional Volatility)	0.76	0.72	0.80	0.81	0.83	0.83	0.80	0.81
Agreeableness (Trust)	0.74	0.76	0.77	0.77	0.80	0.76	0.75	0.75
Open‐Mindedness (Intellectual Curiosity)	0.73	0.76	0.76	0.77	0.79	0.77	0.77	0.73
Extraversion (Energy Level)	0.73	0.76	0.77	0.77	0.78	0.79	0.76	0.75
Open‐Mindedness (Creative Imagination)	0.73	0.77	0.79	0.77	0.77	0.77	0.77	0.71
Agreeableness (Respectfulness)	0.72	0.77	0.80	0.82	0.89	0.79	0.73	0.60
Conscientiousness (Responsibility)	0.71	0.75	0.75	0.82	0.76	0.82	0.77	0.67
Agreeableness (Compassion)	0.66	0.64	0.75	0.77	0.75	0.80	0.73	0.60

*Note: n* = 2424. Each row presents the estimated correlations for value stability over a 2‐year period (ordered by Pearson correlation strength). Quantile correlations highlighted in yellow are significantly weaker than the median (*τ* = 0.5) quantile correlation; none are significantly stronger.

### 3.1 Part 1: Value Stability

The graphs in Figure 1 (with values presented in the order of strength of Pearson correlation) show that value stability varies across the distribution of value importance for almost every value. As expected, value stability tends to be greater at higher levels of value importance and lesser at lower levels. This expected pattern can be seen in 17 of the 20 graphs, in comparison to the Pearson correlations. To illustrate the pattern of relations, observe the plot for the universalism (animals) value (see Figure 1, column 1, row 1). In this case, the Pearson correlation (*r* = 0.75), shown as a gray horizontal line (as it represents a constant degree of association across the distribution), clearly underestimated stability at the higher levels of value importance (e.g., at the 80th percentile the correlation coefficient is 0.90) and overestimated stability at lower levels of value importance (e.g., at the 20th percentile the correlation coefficient is 0.41).

To illustrate the nature of value stability along the distribution of value importance, again consider the universalism (animals) value at the top of Table 1. Its median correlation (i.e., at the 50th percentile) was significantly weaker than the quantile correlation at the 70th and 80th percentile and significantly stronger than the quantile correlations at the 20th, 30th, and 40th percentiles. Overall, Table 1 shows that value stability was greater in 65% of the top quantiles (at the 80th percentile), and 53% of the top three quantiles (at the 60th, 70th, and 80th percentiles) than at the median. Moreover, value stability was lower in 100% of the bottom quantiles (at the 20th percentile) and 80% of the bottom three quantiles (at the 20th, 30th and 40th percentiles) than at the median. Taken together, these results demonstrate the substantial gain in information afforded by examining value stability along the distribution of value importance.

### 3.2 Part 2: Trait Stability

Figure 2 (with traits presented in the order of strength of Pearson correlation) shows that trait‐stability estimates vary little across the distribution of trait strength. Quantile correlation estimates exhibit far less variability across the distribution of trait strength than was observed across the distribution of value importance. There are far fewer significant differences for traits between the respective quantile correlation estimates and the median estimate, with no evidence of an increasing trend in these estimates (see Figure 2). Unlike the general finding for values, trait quantile correlations tended to be stronger at median levels of trait strength than at lower and higher levels. To illustrate the nature of trait stability along the distribution of trait strength, consider extraversion (sociability), which had the highest Pearson correlation (0.83). In Figure 2 (row 1, column 1), it can be seen that the Pearson correlation is comparable to that estimated at both the lower and upper quantiles (20th and 80th percentile), whereas at the middle quantiles (40th, 50th, and 60th percentile) the estimates are higher than the Pearson correlation.

Table 2 indicates that the pattern of trait stability, being greater at the median than at the extremes, is relatively common across traits. Specifically, trait stability was significantly stronger at the median than the top quantile in 13 of the 15 cases, and stronger at the median than the bottom quantile in 7 of the 15 cases.

Taken together, these results suggest a clear difference between the nature of value and trait stability. Specifically, value stability tends to be stronger at higher levels of value importance and weaker at lower levels of value importance. In contrast, trait stability is more consistent across the distribution of trait strength, with a weak tendency to be reduced at the higher and lower extremities of the trait strength distribution.

## 4 Discussion

These findings point to an important and novel difference between personal values and personality traits. Generally, when a value is personally highly important it is much more stable than when it is less important, whereas traits change over time to a similar extent across the distribution of trait strength. This difference was found robustly, despite some systematic links between values and traits that overlap in content (e.g., self‐direction (thought) with open‐mindedness (creativity) *r*_*s*_ = 0.24, *p* < 0.001, at T1 in our sample). While trait stability exhibited much less variability across the distribution of trait strength, there were small but consistent differences. Traits were generally most stable around the median, with small decreases in stability at high levels of trait strength, and sometimes also at low levels of trait strength. These contrasting patterns of stability point to the first empirical evidence of a fundamental difference between values and traits. These patterns suggest that values and traits, even when overlapping in content, are not the same psychological construct.

One explanation for these findings might be based on the nature of values as being motivational in character. Highly important motivations should affect behavior across different situations, whereas a less important motivation is likely to be more easily thwarted by situational factors. As values are cognitions (e.g., Schwartz 1992), and according to our knowledge on cognitive schemas (see Bardi and Goodwin 2011), with every value‐expressive behavior enacted, the value itself may be strengthened, making it even less amenable to change. This should result in highly important values becoming more strongly embedded in one's self‐concept and thus also more likely to withstand situational changes. In contrast, values that matter less, and are therefore less often expressed by concrete behavior, may fluctuate more as a function of life conditions, and they may also fluctuate more momentarily depending on how the individual feels at a given time.

It is also important to note that there were some exceptions to the patterns of value stability. The two benevolence values (caring and dependability) appeared to show a similar pattern to traits, being slightly less stable at the extremes than at the median. In fact, the graph of benevolence (dependability) (see Figure 1) is very similar to the graph of trait conscientiousness (responsibility) (see Figure 2) and the two substantially overlap in content. The two security values (societal and personal) also showed a less consistent pattern, especially societal security. Interestingly, these were the same values that did not exhibit a clear pattern of increasing value‐behavior relations with increasing value importance in Lee et al. (2022). In their paper, they argued that benevolence values are subject to normative pressures that encourage people to behave consistently with these values, no matter their individual value importance (Lee et al. 2022). This might also be a possible explanation for the pattern of stability for security values, as benevolence and security values were the most important values in our sample, overall (see Table S1). As these values are highly normative, it is possible that behaving according to them strengthens schemas of norms rather than personal value importance, as they may be linked more to norms than to values, and hence their stability does not depend on their personal importance.

Interestingly, when we examine Pearson's correlations in our data, a difference in stability between values and traits does appear to exist, with the average correlation for the 15 traits (*r* = 0.75) being higher than the average correlation for the 20 values (*r* = 0.55; *z* = 13.109, *p* < 0.001).^3^ However, when quantile correlations are compared at the 80th percentile, the average correlation for the 15 traits (*r* = 0.74) was no higher than the average correlation for the 20 values (*r* = 0.77). Further, it is clear from the analysis that estimates based on average relations underestimate the stability of values at higher levels of value importance and overestimate stability at lower levels of value importance. For instance, the observed Pearson's correlation for power (resources) value stability was 0.57; however, the quantile correlation coefficient at the 80th percentile of the value importance distribution was 0.92, in contrast to 0.25 at the 20th percentile. Thus, meaningful knowledge about value stability is likely to be masked when employing commonly used linear techniques (e.g., Pearson correlations, ordinary least squares [OLS] regression, and mean‐level difference models); however, these models may accurately reflect the stability of traits and of associations between traits and other related phenomena.

The findings of the current study also have specific implications for understanding value change over the life course. For instance, major crises (e.g., COVID‐19; Daniel et al. 2022), life‐changing events (Bardi et al. 2009), and emigrating to another country (Bardi et al. 2014; Lönnqvist, Jasinskaja‐Lahti, and Verkasalo 2011) have all been found to be accompanied by value change in adults. Future research should examine whether these changes are dependent on value importance. Specifically, we might expect highly important values to be less malleable, both in response to external events and to biological and psychological maturation. It may also be important to examine this phenomenon in adolescents, given that adults exhibit less change in values than younger people in general (Benish‐Weisman, Daniel, and Knafo‐Noam 2017).

Future research could also examine and compare the influence of value importance on value stability within and between samples. For instance, do specific groups who are relatively low on a value of interest (e.g., social work students are lower on achievement and power values than business school students; Arieli, Sagiv, and Cohen‐Shalem 2016) exhibit less value stability and also weaker value‐behavior relations than groups that are relatively high on that value? In the case of the above example, research could investigate whether social work students who are relatively higher on achievement and power value than their peers show similar patterns of value stability to business school students. Importantly however, in order to do this, researchers will need to consider the way they measure the variables of interest to ensure that there is sufficient continuity in the measurement scales used to facilitate the use of quantile techniques.

Further, if values held as highly important are highly stable, we might also expect that they will be commensurately difficult to change through deliberate interventions. Researchers attempting to change values, or related behavior, may need to take into account how important the value is to the target. There is some potential evidence for this, as interventions targeting benevolence values have been found to produce value change (Arieli, Grant, and Sagiv 2014), and we show that benevolence values do not become more stable at higher levels of importance, as other values do. Attaining a more complete understanding of the interplay between value importance, value stability, and value change will enable us to better craft and target value‐based interventions.

Results may also have implications for understanding value‐behavior relations. Values that are highly important have been found to be more strongly related to present (Lee et al. 2022) and future behavior (Lake et al. 2024) and also appear to be commensurately more stable. This lends weight to the idea that value‐behavior relations persist over extended periods of time, which may be due to repeated value‐expressive behavior working reciprocally to further strengthen the value (see Bardi and Goodwin 2011). Future research could model the longitudinal interactions between values and behavior, and investigate whether other individual (e.g., genetic) and environmental (e.g., cultural/societal) factors underly and influence these patterns of relations.

### 4.1 Limitations

There are a number of limitations of the present study that must be noted, including the use of self‐reports for the measurement of values and traits. However, although values and traits were both measured as self‐reports, it is possible to argue that for these internal variables, the individual has the best access to their inner thoughts and feelings, and therefore self‐reports may be the most valid ways of assessment for values and traits. Nonetheless, future research may attempt to replicate our findings using non‐self‐report measures, such as acquaintance reports (e.g., Dobewall et al. 2014).

Consideration of the instruments used also raises the question of whether measures employed to elicit values and traits produced equally reliable estimates of the underlying constructs and whether these differences may account for the results. For instance, it is possible that the BWVr task may be more (or less) difficult for participants than the BFI‐2 rating scale task. It is also possible that there may be more (or less) measurement error in the BWVr than BFI‐2 responses. In best‐worst scaling, we would expect more measurement error to be found around the midpoint of the scale, as less information is elicited about values that are neither important nor unimportant. It is also possible for participants to have a similar score for a value that was never chosen as most or least important in any set, as for another that was chosen as most important in one value set and least important in another set, potentially because they were not confident of their responses. However, our results tend to show increasing value stability from the lowest to the highest quantile of value importance, rather than a dip in the middle. On this basis, measurement error is unlikely to be a driver of the pattern of results we found.

Alternatively, if it were the case that participants are only confident of their responses for the few values they hold as most important, and respond inconsistently to the rest, we would expect a steeper “drop‐off” in stability after the upper quantiles of value importance and consistently (low) stability across the remaining quantiles. In contrast, we tend to see a steady decline in stability across decreasing quantiles of value importance in most cases. Further, this explanation does not account for the characteristic differences in stability observed with certain values (e.g., benevolence and security values), and similarities between values and traits when content overlaps (e.g., the benevolence (dependability) value and the conscientiousness (responsibility) trait). Additionally, the fact that respondents tend to rate most rating scale value items from “somewhat important” to “very important” (Schwartz and Bardi 2001), despite the fact that values exist as ordered priorities within individuals, suggests that people may be less able to differentiate which values are most important to them unless presented with direct comparisons.

It is also possible that quantile regression techniques are more suited to best‐worst scaling than rating scales. While further research is needed to examine the stability of traits and values using other types of measurement, we provide some evidence that differences in the patterns of stability identified with quantile correlations are not scale‐dependent, by analyzing values data collected with different instruments in the same sample. Supporting Information, Appendix E provides an example that shows (a) quantile correlations between neighboring values are higher at higher levels of importance than at lower levels, using data elicited with both best‐worst and rating scales, and (b) there is greater stability across different scale types at higher levels of value importance than at lower levels. However, as previously mentioned, more research is needed to further explore the findings of the current study using different value and trait measures (e.g., peer reports), to see whether the results replicate across methods and samples.

Sampling limitations must also be noted. The sample examined was large and diverse across a range of socio‐demographics including age, socio‐economic status, and education level (see Lee et al. 2019). However, it should be acknowledged that these findings are largely reliant on “WEIRD” participants (Henrich, Heine, and Norenzayan 2010) and that there was significant attrition between T1 (2017) and T2 (2019) on a variety of values and traits. These issues could set some limits to generalizability; however, it should also be noted that the large sample size increased the chances of finding significant differences between subgroups and that the pattern of results from the attrition analyses does not seem to be related to differences in quantile correlations. Further cross‐cultural and comparative work across a wider variety of cultural backgrounds may serve to either strengthen the present findings or highlight further complexities. For instance, those values of greater importance to a culture may be more stable in its citizens, especially in tight cultures, where there are strong social norms and intolerance for deviation from them (Elster and Gelfand 2021). We might also expect to observe differences in the stability of values and traits in less individualistic societies, or where there is stronger cultural (or even political) pressure to conform. The present findings do not address such potential cultural variations.

## 5 Conclusion

The present study suggests a key difference between personal values and personality traits. While values exhibit clearly non‐linear patterns of stability, trait stability appears to be either linear or slightly decreased at its extremes. In general, more important values tend to remain more stable over time, and highly important values may remain as stable as traits. Results have implications not only for the future of values and personality research but also for the understanding of value development and the links between values and behavior over time.

The consistent difference found between value and trait stability provides clear evidence that the two are not the same psychological variable simply measured in a different way. Instead, the present findings strengthen the idea that values, as broad motivational life goals, constitute core elements of personality when they are highly prioritized, whereas traits are overall descriptions of individuals, that are core elements of personality at any level of trait strength. Thus, the difference between values and traits is not merely semantic, but is more meaningful and has important consequences. This paper therefore provides an important step forward in the quest to better understand how these central aspects of personality can be similar in content, and yet fundamentally different in nature.

## Author Contributions

Joshua Lake was responsible for drafting the original manuscript. All authors contributed to conceptualization. Julie A. Lee and Anat Bardi contributed to the funding acquisition. Julie A. Lee was responsible for data collection. Joshua Lake and Julie A. Lee were responsible for the data analysis. All authors contributed to reviewing and editing. Joanne Sneddon and Julie A. Lee provided project supervision.

## Ethics Statement

Data collection was approved by The University of Western Australia Human Research Ethics Committee (RA/4/1/8647).

## Conflicts of Interest

The authors declare no conflicts of interest.

## Supporting information


Data S1.

## Data Availability

All data and syntax used to produce these results can be accessed at the following link: https://osf.io/s2c74/?view_only=cd0b3a23376344fc87ab34f837ee0ee4.

## Endnotes

^1^
Similar results were obtained when comparing the first (2017) and second (2018) waves; we have chosen to present analysis over the longer time period.

^2^
To paraphrase Koenker and Hallock (2001), a respondent has a value importance score at the *τ*th quantile of a sample if they have a higher value importance score than the proportion *τ* of the sample or a lower score than the proportion (1−*τ*).

^3^
In this case, a comparison of correlations from independent samples (per Eid, Gollwitzer, and Schmidt 2011, 547; single‐sided test) was used, as there was no common variable; however, it should be noted that the sample is overlapping.

## References

[jopy12979-bib-0001] Arieli, S. , A. M. Grant , and L. Sagiv . 2014. “Convincing Yourself to Care About Others: An Intervention for Enhancing Benevolence Values.” Journal of Personality 82, no. 1: 15–24. 10.1111/jopy.12029. 23301825

[jopy12979-bib-0002] Arieli, S. , L. Sagiv , and E. Cohen‐Shalem . 2016. “Values in Business Schools: The Role of Self‐Selection and Socialization.” Academy of Management Learning & Education 15, no. 3: 493–507. 10.5465/amle.2014.0064.

[jopy12979-bib-0003] Bardi, A. , K. E. Buchanan , R. Goodwin , L. Slabu , and M. Robinson . 2014. “Value Stability and Change During Self‐Chosen Life Transitions: Self‐Selection Versus Socialization Effects.” Journal of Personality and Social Psychology 106, no. 1: 131–147. 10.1037/a0034818. 24219783

[jopy12979-bib-0004] Bardi, A. , and R. Goodwin . 2011. “The Dual Route to Value Change: Individual Processes and Cultural Moderators.” Journal of Cross‐Cultural Psychology 42, no. 2: 271–287. 10.1177/0022022110396916.

[jopy12979-bib-0005] Bardi, A. , J. A. Lee , N. Hofmann‐Towfigh , and G. Soutar . 2009. “The Structure of Intraindividual Value Change.” Journal of Personality and Social Psychology 97, no. 5: 913–929. 10.1037/a0016617. 19857010

[jopy12979-bib-0006] Baumert, A. , M. Schmitt , M. Perugini , et al. 2017. “Integrating Personality Structure, Personality Process, and Personality Development.” European Journal of Personality 31: 503–528. 10.1002/per2115.

[jopy12979-bib-0007] Benish‐Weisman, M. , E. Daniel , and A. Knafo‐Noam . 2017. “The Relations Between Values and Aggression: A Developmental Perspective.” In Values and Behavior: Taking a Cross‐Cultural Perspective, edited by S. Roccas and L. Sagiv , 97–114. Cham, Switzerland: Springer International.

[jopy12979-bib-0008] Bleidorn, W. , T. Schwaba , A. Zheng , et al. 2022. “Personality Stability and Change.” Psychological Bulletin 148, no. 7–8: 588–619. 10.1037/bul0000365. 35834197

[jopy12979-bib-0010] Bouchard, T. J., Jr. , and M. McGue . 2003. “Genetic and Environmental Influences on Human Psychological Differences.” Journal of Neurobiology 54, no. 1: 4–45. 10.1002/neu.10160. 12486697

[jopy12979-bib-0011] Caprara, G. V. , S. Schwartz , C. Capanna , M. Vecchione , and C. Barbaranelli . 2006. “Personality and Politics: Values, Traits, and Political Choice.” Political Psychology 27, no. 1: 1–28. 10.1111/j.1467-9221.2006.00457.x.

[jopy12979-bib-0012] Choi, J. E. , and D. W. Shin . 2022. “Quantile Correlation Coefficient: A New Tail Dependence Measure.” Statistical Papers 63: 1075–1104. 10.1007/s00362-021-01268-7.

[jopy12979-bib-0013] Costello, T. H. , S. M. Bowes , S. T. Stevens , I. D. Waldman , A. Tasimi , and S. O. Lilienfeld . 2022. “Clarifying the Structure and Nature of Left‐Wing Authoritarianism.” Journal of Personality and Social Psychology 122, no. 1: 135–170. 10.1037/pspp0000341. 34383522

[jopy12979-bib-0014] Damian, R. I. , M. Spengler , A. Sutu , and B. W. Roberts . 2019. “Sixteen Going on Sixty‐Six: A Longitudinal Study of Personality Stability and Change Across 50 Years.” Journal of Personality and Social Psychology 117, no. 3: 674–695. 10.1037/pspp0000210. 30113194

[jopy12979-bib-0055] Daniel, E. , A. Bardi , R. Fischer , M. Benish‐Weisman , and J. A. Lee . 2022. “Changes in Personal Values in Pandemic Times.” Social Psychological and Personality Science 13, no. 2: 572–582. 10.1177/19485506211024026

[jopy12979-bib-0015] Deary, I. 2009. “The Trait Approach to Personality.” In The Cambridge Handbook of Personality Psychology, edited by P. J. Corr and G. Matthews , 89–109. Cambridge, UK: Cambridge University Press.

[jopy12979-bib-0016] Dobewall, H. , and T. Aavik . 2016. “Rank‐Order Consistency and Profile Stability of Self‐ and Informant‐Reports of Personal Values in Comparison to Personality Traits.” Journal of Individual Differences 37, no. 1: 40–48. 10.1027/1614-0001/a000186.

[jopy12979-bib-0017] Dobewall, H. , T. Aavik , K. Konstabel , S. H. Schwartz , and A. Realo . 2014. “A Comparison of Self‐Other Agreement in Personal Values Versus the Big Five Personality Traits.” Journal of Research in Personality 50: 1–10. 10.1016/j.jrp.2014.01.004.

[jopy12979-bib-0018] Eid, M. , M. Gollwitzer , and M. Schmidt . 2011. Statistik und Forschungsmethoden Lehrbuch. Weinheim, Germany: Beltz.

[jopy12979-bib-0019] Elster, A. , and M. J. Gelfand . 2021. “When Guiding Principles Do Not Guide: The Moderating Effects of Cultural Tightness on Value‐Behavior Links.” Journal of Personality 89, no. 2: 325–337. 10.1111/jopy.12584. 32772368

[jopy12979-bib-0020] Fetvadjiev, V. H. , and J. He . 2019. “The Longitudinal Links of Personality Traits, Values, and Well‐Being and Self‐Esteem: A Five‐Wave Study of a Nationally Representative Sample.” Journal of Personality and Social Psychology 117, no. 2: 448–464. 10.1037/pspp0000212. 30394770

[jopy12979-bib-0021] Fischer, R. , and D. Boer . 2015. “Motivational Basis of Personality Traits: A Meta‐Analysis of Value‐Personality Correlations.” Journal of Personality 83, no. 5: 491–510. 10.1111/jopy.12125. 25142346

[jopy12979-bib-0022] Gong, Z. 2016. “Estimation of Sample Size and Power for Quantile Regression.” Doctoral diss., Queen's University Kingston, ON, Canada.

[jopy12979-bib-0023] Henrich, J. , S. J. Heine , and A. Norenzayan . 2010. “The Weirdest People in the World?” Behavioural and Brain Sciences 33, no. 2–3: 61–83. 10.1017/S0140525X0999152X. 20550733

[jopy12979-bib-0024] Hopwood, C. J. , M. B. Donnellan , D. M. Blonigen , et al. 2011. “Genetic and Environmental Influences on Personality Trait Stability and Growth During the Transition to Adulthood: A Three‐Wave Longitudinal Study.” Journal of Personality and Social Psychology 100, no. 3: 545–556. 10.1037/a0022409. 21244174 PMC3058678

[jopy12979-bib-0025] Huang, Q. , H. Zhang , J. Chen , and M. He . 2017. “Quantile Regression Models and Their Applications: A Review.” Journal of Biometrics and Biostatistics 8, no. 3: 1–6. 10.4172/2155-6180.1000354.30555734

[jopy12979-bib-0026] Hulland, J. , H. Baumgartner , and K. M. Smith . 2018. “Marketing Survey Research Best Practices: Evidence and Recommendations From a Review of JAMS Articles.” Journal of the Academy of Marketing Science 46, no. 1: 92–108. 10.1007/s11747-017-0532-y.

[jopy12979-bib-0027] John, O. , and S. Srivastava . 1999. “The Big Five Trait Taxonomy: History, Measurement, and Theoretical Perspectives.” In Handbook of Personality: Theory and Research, edited by L. A. Pervin and O. P. John , 102–138. New York, NY: Guilford Press.

[jopy12979-bib-0028] Koenker, R. , and G. Bassett . 1978. “Regression Quantiles.” Econometrica 46, no. 1: 33–50. 10.2307/1913643.

[jopy12979-bib-0029] Koenker, R. , and K. F. Hallock . 2001. “Quantile Regression.” Journal of Economic Perspectives 15, no. 4: 143–156. 10.1257/jep.15.4.143.

[jopy12979-bib-0030] Lake, J. , J. Sneddon , A. Bardi , and J. A. Lee . 2024. “How Far Into the Future Can Values Predict Behavior? It Depends on Value Importance.” Social Psychological and Personality Science. Published ahead of print. 10.1177/19485506241233646.

[jopy12979-bib-0031] Lee, J. , U. Evers , J. Sneddon , O. Rahn , and S. Schwartz . 2019. What Do We Value? How Our Values Influence Everyday Behavior. Perth, Australia: Centre for Human and Cultural Values. http://whatdowevalue.com.au/wp‐content/uploads/2019/03/Values‐Report‐Final‐2019‐SM.pdf.

[jopy12979-bib-0032] Lee, J. , J. N. Sneddon , T. M. Daly , S. H. Schwartz , G. N. Soutar , and J. Louviere . 2019. “Testing and Extending Schwartz Refined Value Theory Using a Best‐Worst Scaling Approach.” Assessment 26, no. 2: 166–180. 10.1177/1073191116683799. 30740999

[jopy12979-bib-0033] Lee, J. A. , A. Bardi , P. Gerrans , et al. 2022. “Are Value‐Behavior Relations Stronger Than Previously Thought? It Depends on Value Importance.” European Journal of Personality 36, no. 2: 133–148. 10.1177/08902070211002965.

[jopy12979-bib-0034] Lee, J. A. , G. Soutar , and J. Louviere . 2008. “The Best–Worst Scaling Approach: An Alternative to Schwartz's Values Survey.” Journal of Personality Assessment 90, no. 4: 335–347. 10.1080/00223890802107925. 18584442

[jopy12979-bib-0035] Löckenhoff, C. E. , A. Terracciano , N. S. Patriciu , W. W. Eaton , and P. T. Costa Jr. 2009. “Self‐Reported Extremely Adverse Life Events and Longitudinal Changes in Five‐Factor Model Personality Traits in an Urban Sample.” Journal of Traumatic Stress 22, no. 1: 53–59. 10.1002/jts.20385. 19230009 PMC2761831

[jopy12979-bib-0036] Lönnqvist, J. E. , I. Jasinskaja‐Lahti , and M. Verkasalo . 2011. “Personal Values Before and After Migration: A Longitudinal Case Study on Value Change in Ingrian–Finnish Migrants.” Social Psychology and Personality Science 2: 584–591. 10.1177/1948550611402362.

[jopy12979-bib-0037] McCrae, R. R. . 2009. “The Five‐Factor Model of Personality Traits: Consensus and Controversy.” In The Cambridge Handbook of Personality Psychology, edited by P. J. Corr and G. Matthews , 148. Cambridge, UK: Cambridge University Press.

[jopy12979-bib-0038] McCrae, R. R. , and P. T. Costa Jr. . 2008. “The Five‐Factor Theory of Personality.” In Handbook of Personality: Theory and Research, edited by O. P. John , R. W. Robins , and L. A. Pervin , 3rd ed., 159–180. New York, NY: Guilford Press.

[jopy12979-bib-0039] Parks‐Leduc, L. , G. Feldman , and A. Bardi . 2015. “Personality Traits and Personal Values: A Meta‐Analysis.” Personality and Social Psychology Review 19, no. 1: 3–29. 10.1177/1088868314538548. 24963077

[jopy12979-bib-0040] Pozzebon, J. A. , and M. C. Ashton . 2009. “Personality and Values as Predictors of Self‐ and Peer‐Reported Behavior.” Journal of Individual Differences 30, no. 3: 122–129. 10.1027/1614-0001.30.3.122.

[jopy12979-bib-0041] Roberts, B. W. , and L. B. Nickel . 2017. “A Critical Evaluation of the Neo‐Socioanalytic Model of Personality.” In Personality Development Across the Lifespan, edited by J. Specht , 157–177. Cambridge, MA: Academic Press.

[jopy12979-bib-0042] Roccas, S. , L. Sagiv , S. H. Schwartz , and A. Knafo . 2002. “The Big Five Personality Factors and Personal Values.” Personality and Social Psychology Bulletin 28, no. 6: 789–801. 10.1177/0146167202289008.

[jopy12979-bib-0043] Schermer, J. A. , P. A. Vernon , G. R. Maio , and K. L. Jang . 2011. “A Behavior Genetic Study of the Connection Between Social Values and Personality.” Twin Research and Human Genetics 14, no. 3: 233–239. 10.1375/twin.14.3.233. 21623653

[jopy12979-bib-0044] Schwartz, S. H. 1992. “Universals in the Content and Structure of Values: Theoretical Advances and Empirical Tests in 20 Countries.” In Advances in Experimental Social Psychology, Vol. 25, edited by M. P. Zanna , 1–65. Cambridge, MA: Academic Press. 10.1016/S0065-2601(08)60281-6.

[jopy12979-bib-0045] Schwartz, S. H. 2012. “An Overview of the Schwartz Theory of Basic Values.” Online Readings in Psychology and Culture 2, no. 1: 1–20. 10.9707/2307-0919.1116.

[jopy12979-bib-0046] Schwartz, S. H. , and A. Bardi . 2001. “Value Hierarchies Across Cultures: Taking a Similarities Perspective.” Journal of Cross‐Cultural Psychology 32, no. 3: 268–290. 10.1177/0022022101032003002.

[jopy12979-bib-0047] Skimina, E. , and J. Cieciuch . 2020. “Explaining Everyday Behaviours and Situational Context by Personality Metatraits and Higher‐Order Values.” European Journal of Personality 34: 29–59. 10.1002/per2230.

[jopy12979-bib-0048] Skimina, E. , J. Cieciuch , and W. Strus . 2021. “Traits and Values as Predictors of the Frequency of Everyday Behavior: Comparison Between Models and Levels.” Current Psychology 40, no. 1: 133–153. 10.1007/s12144-018-9892-9.

[jopy12979-bib-0049] Soto, C. J. , and O. John . 2017. “The Next Big Five Inventory (BFI‐2): Developing and Assessing a Hierarchical Model With 15 Facets to Enhance Bandwidth, Fidelity, and Predictive Power.” Journal of Personality and Social Psychology 113, no. 1: 117–143. 10.1037/pspp0000096. 27055049

[jopy12979-bib-0050] Twito, L. , and A. Knafo‐Noam . 2020. “Beyond Culture and the Family: Evidence From Twin Studies on the Genetic and Environmental Contribution to Values.” Neuroscience & Biobehavioral Reviews 112: 135–143. 10.1016/j.neubiorev.2019.12.029. 31917161

[jopy12979-bib-0051] Vecchione, M. , G. Alessandri , S. Roccas , and G. V. Caprara . 2019. “A Look Into the Relationship Between Personality Traits and Basic Values: A Longitudinal Investigation.” Journal of Personality 87, no. 2: 413–427. 10.1111/jopy.12399. 29806202

[jopy12979-bib-0052] Vecchione, M. , S. H. Schwartz , E. Davidov , J. Cieciuch , G. Alessandri , and G. Marsicano . 2020. “Stability and Change of Basic Personal Values in Early Adolescence: A 2‐Year Longitudinal Study.” Journal of Personality 88, no. 3: 447–463. 10.1111/jopy.12502. 31402448

[jopy12979-bib-0053] Zou, G. Y. 2007. “Toward Using Confidence Intervals to Compare Correlations.” Psychological Methods 12, no. 4: 399–413. 10.1037/1082-989X.12.4.399. 18179351

