# Supplementary material for: A Fundamental Difference in the Nature of Personal Values and Personality Traits Revealed Through Different Patterns of Stability Across Their Distributions
Source: J Pers. 2024 Oct 4;93(4):830–44. doi: 10.1111/jopy.12979 (PMC12224552; doi:10.1111/jopy.12979)
Supplement: Supplementary file 1 — Data S1. [file JOPY-93-830-s001.docx]

**Supplementary Materials**

**Page**

Appendix A: Descriptive statistics 2

Table S1: Values descriptive statistics 2

Table S2: Traits descriptive statistics 3

Table S3: Attrition analyses for values 4

Table S4: Attrition analyses for traits 5

Appendix B: Adequacy of sample size 6

Table S5: Minimum sample size requirements 6

Appendix C: Reliability testing 7

Table S6: Times a participant’s most important value was chosen 7

Table S7: Time 1 trait items rotated component matrix (acquiescence removed) 9

Table S8: Time 2 trait items rotated component matrix (acquiescence removed) 11

Table S9: Trait alphas 13

Appendix D: Quantile correlation tables with confidence estimates 14

Table S10: Quantile correlation results with confidence estimates: personal values 14

Table S11: Quantile correlation results with confidence estimates: personality traits 16

Appendix E: Comparing best-worst and rating scale measures of values 18

Table S12: Scale comparison: values descriptive statistics 20

Figure S1: Quantile correlation results: universalism values (within and between alternative measures) 22

Figure S2: Quantile correlation results: universalism values (SVBWS values jittered) 23

**Appendix A: Descriptive Statistics**

**Table S1**

*Values descriptive statistics*

|  | Time 1 (2017) | | | | | Time 2 (2019) | | | |
| --- | --- | --- | --- | --- | --- | --- | --- | --- | --- |
| Personal values (refined) | Mean | *SD* | Min. | Max. | Mean | | *SD* | Min. | Max. |
| Self-Direction (thought) | 1.54 | 0.70 | 0.25 | 4.00 | 1.55 | | 0.68 | 0.25 | 4.00 |
| Self-Direction (action) | 1.70 | 0.84 | 0.40 | 4.00 | 1.77 | | 0.87 | 0.25 | 4.00 |
| Stimulation | 1.32 | 0.73 | 0.25 | 4.00 | 1.30 | | 0.73 | 0.25 | 4.00 |
| Hedonism | 1.49 | 0.80 | 0.25 | 4.00 | 1.44 | | 0.78 | 0.25 | 4.00 |
| Achievement | 0.79 | 0.56 | 0.25 | 4.00 | 0.78 | | 0.57 | 0.25 | 4.00 |
| Power (resources) | 0.73 | 0.62 | 0.25 | 4.00 | 0.73 | | 0.60 | 0.25 | 4.00 |
| Power (dominance) | 0.60 | 0.38 | 0.25 | 4.00 | 0.60 | | 0.37 | 0.25 | 3.40 |
| Face | 0.84 | 0.47 | 0.25 | 4.00 | 0.85 | | 0.47 | 0.25 | 3.40 |
| Security (personal) | 1.88 | 0.84 | 0.25 | 4.00 | 1.89 | | 0.84 | 0.40 | 4.00 |
| Security (societal) | 2.29 | 0.89 | 0.40 | 4.00 | 2.24 | | 0.86 | 0.40 | 4.00 |
| Tradition | 0.98 | 0.69 | 0.25 | 4.00 | 0.99 | | 0.73 | 0.25 | 4.00 |
| Conformity (rules) | 1.32 | 0.75 | 0.25 | 4.00 | 1.30 | | 0.74 | 0.25 | 4.00 |
| Conformity (interpersonal) | 0.98 | 0.55 | 0.25 | 4.00 | 0.95 | | 0.53 | 0.25 | 4.00 |
| Humility | 1.13 | 0.60 | 0.25 | 4.00 | 1.19 | | 0.63 | 0.25 | 4.00 |
| Benevolence (dependability) | 2.57 | 1.00 | 0.25 | 4.00 | 2.54 | | 1.01 | 0.25 | 4.00 |
| Benevolence (caring) | 2.48 | 0.91 | 0.55 | 4.00 | 2.43 | | 0.91 | 0.40 | 4.00 |
| Universalism (concern) | 1.94 | 0.93 | 0.25 | 4.00 | 1.92 | | 0.94 | 0.55 | 4.00 |
| Universalism (nature) | 1.51 | 0.85 | 0.25 | 4.00 | 1.65 | | 0.91 | 0.25 | 4.00 |
| Universalism (animals) | 1.48 | 0.93 | 0.25 | 4.00 | 1.53 | | 0.96 | 0.25 | 4.00 |
| Universalism (tolerance) | 1.88 | 0.88 | 0.25 | 4.00 | 1.81 | | 0.86 | 0.25 | 4.00 |

*Note*. *n* = 2,875.

**Table S2**

*Traits descriptive statistics*

|  | Time 1 (2017) | | | | Time 2 (2019) | | | |
| --- | --- | --- | --- | --- | --- | --- | --- | --- |
| Personality trait (facet) | Mean | *SD* | Min. | Max. | Mean | *SD* | Min. | Max. |
| Extraversion (Sociability) | -0.14 | 0.91 | -2.00 | 2.00 | -0.17 | 0.96 | -2.00 | 2.00 |
| Extraversion (Assertiveness) | -0.01 | 0.81 | -2.00 | 2.00 | -0.09 | 0.81 | -2.00 | 2.00 |
| Extraversion (Energy) | 0.23 | 0.80 | -2.00 | 2.00 | 0.21 | 0.81 | -2.00 | 2.00 |
| Agreeableness (Compassion) | 0.95 | 0.70 | -2.00 | 2.00 | 0.96 | 0.70 | -2.00 | 2.00 |
| Agreeableness (Respectfulness) | 1.09 | 0.68 | -1.75 | 2.00 | 1.09 | 0.67 | -1.50 | 2.00 |
| Agreeableness (Trust) | 0.38 | 0.74 | -2.00 | 2.00 | 0.41 | 0.74 | -2.00 | 2.00 |
| Conscientiousness (Organization) | 0.85 | 0.85 | -2.00 | 2.00 | 0.86 | 0.85 | -2.00 | 2.00 |
| Conscientiousness (Productiveness) | 0.73 | 0.78 | -2.00 | 2.00 | 0.73 | 0.79 | -2.00 | 2.00 |
| Conscientiousness (Responsibility) | 0.88 | 0.70 | -2.00 | 2.00 | 0.89 | 0.69 | -2.00 | 2.00 |
| Negative Emotionality (Anxiety) | 0.17 | 0.90 | -2.00 | 2.00 | 0.13 | 0.95 | -2.00 | 2.00 |
| Negative Emotionality (Depression) | -0.37 | 0.92 | -2.00 | 2.00 | -0.40 | 0.95 | -2.00 | 2.00 |
| Negative Emotionality (Emotional Volatility) | -0.32 | 0.90 | -2.00 | 2.00 | -0.35 | 0.90 | -2.00 | 2.00 |
| Open-Mindedness (Intellectual Curiosity) | 0.64 | 0.69 | -1.75 | 2.00 | 0.61 | 0.73 | -2.00 | 2.00 |
| Open-Mindedness (Aesthetic Sensitivity) | 0.24 | 0.86 | -2.00 | 2.00 | 0.21 | 0.89 | -2.00 | 2.00 |
| Open-Mindedness (Creative Imagination) | 0.56 | 0.76 | -2.00 | 2.00 | 0.53 | 0.79 | -2.00 | 2.00 |

*Note*. *n* = 2,424.

**Table S3**

*Attrition analyses for values*

| Personal values (refined) | Lost to Attrition | Panel completed | *F*-value | *p*-value |
| --- | --- | --- | --- | --- |
| Self-Direction (thought) | 1.50 | 1.54 | 6.37 | .01 |
| Self-Direction (action) | 1.72 | 1.70 | 1.02 | .31 |
| Stimulation | 1.42 | 1.32 | 32.91 | .00 |
| Hedonism | 1.63 | 1.49 | 51.45 | .00 |
| Achievement | 1.03 | 0.79 | 224.72 | .00 |
| Power (resources) | 0.80 | 0.73 | 21.58 | .00 |
| Power (dominance) | 0.66 | 0.60 | 35.69 | .00 |
| Face | 0.88 | 0.84 | 10.09 | .00 |
| Security (personal) | 1.83 | 1.88 | 8.35 | .00 |
| Security (societal) | 2.18 | 2.29 | 25.51 | .00 |
| Tradition | 0.98 | 0.98 | 0.08 | .78 |
| Conformity (rules) | 1.20 | 1.32 | 49.48 | .00 |
| Conformity (interpersonal) | 0.99 | 0.99 | 0.01 | .94 |
| Humility | 1.13 | 1.13 | 0.12 | .73 |
| Benevolence (dependability) | 2.40 | 2.57 | 48.88 | .00 |
| Benevolence (caring) | 2.38 | 2.48 | 19.50 | .00 |
| Universalism (concern) | 1.90 | 1.94 | 2.89 | .09 |
| Universalism (nature) | 1.51 | 1.51 | 0.03 | .85 |
| Universalism (animals) | 1.45 | 1.48 | 2.38 | .12 |
| Universalism (tolerance) | 1.87 | 1.88 | 0.21 | .65 |
| Age | 43.18 | 52.91 | 757.21 | .00 |

*Note*. *n* = 2,875 completed and 4,578 lost to attrition between 2017 and 2019.

**Table S4**

*Attrition analyses for traits*

| Personality trait (facet) | Lost to Attrition | Panel completed | *F*-value | *p*-value |
| --- | --- | --- | --- | --- |
| Extraversion (Sociability) | -0.14 | -0.14 | 0.00 | .95 |
| Extraversion (Assertiveness) | -0.01 | -0.01 | 0.00 | .98 |
| Extraversion (Energy) | 0.20 | 0.23 | 2.40 | .12 |
| Agreeableness (Compassion) | 0.80 | 0.95 | 73.67 | .00 |
| Agreeableness (Respectfulness) | 0.93 | 1.09 | 82.48 | .00 |
| Agreeableness (Trust) | 0.28 | 0.38 | 31.60 | .00 |
| Conscientiousness (Organization) | 0.64 | 0.85 | 88.59 | .00 |
| Conscientiousness (Productiveness) | 0.53 | 0.73 | 108.39 | .00 |
| Conscientiousness (Responsibility) | 0.68 | 0.88 | 130.02 | .00 |
| Negative Emotionality (Anxiety) | 0.27 | 0.17 | 18.48 | .00 |
| Negative Emotionality (Depression) | -0.23 | -0.37 | 37.07 | .00 |
| Negative Emotionality (Emotional Volatility) | -0.13 | -0.32 | 69.11 | .00 |
| Open-Mindedness (Intellectual Curiosity) | 0.63 | 0.64 | 1.14 | .28 |
| Open-Mindedness (Aesthetic Sensitivity) | 0.26 | 0.24 | 0.51 | .48 |
| Open-Mindedness (Creative Imagination) | 0.51 | 0.56 | 5.87 | .02 |
| Age | 44.41 | 52.98 | 506.87 | .00 |

*Note*. *n* = 2,424 completed and 4,223 lost to attrition between 2017 and 2019.

**Appendix B: Adequacy of Sample Size**

We used the R function power.rq.test developed by Gong (2016) to calculate minimum samples sizes for our study. We calculated minimum required sample sizes, based on the size of the standard deviation of the variables in our sample, for different levels of correlation (*r*) and at different quantiles (τ ), assuming a power value of .80 and a *p*-value of .05. As sample size depends on the standard deviation of the variables of interest, we show in Table S5 four cases, with standard deviations in rating scale data ranging from .25 to .75 (in rows). Sample size requirements also differ depending on the strength of the relationships (correlation) and on the quantiles being examined. To provide insight, we show in Table S3 the results for four levels of correlations (.2, .3, .4, .5) and five quantiles (i.e., τ = .2, .4, .5, .6, and .8), and indicate the required minimum sample size for our study, for a power value of .80 and a *p*-value of 0.05, for selected levels.

**Table S5**

*Minimum sample size requirements*

| *SD* | *r* | τ = .2 | τ = .4 | τ = .5 | τ = .6 | τ = .8 |
| --- | --- | --- | --- | --- | --- | --- |
| 0.25 | .2 | 5787 | 4558 | 4453 | 4558 | 5787 |
|  | .3 | 2573 | 2027 | 1981 | 2027 | 2573 |
|  | .4 | 1448 | 1141 | 1115 | 1141 | 1448 |
|  | .5 | 928 | 731 | 715 | 731 | 928 |
| 0.50 | .2 | 1448 | 1141 | 1115 | 1141 | 1448 |
|  | .3 | 645 | 509 | 497 | 509 | 645 |
|  | .4 | 364 | 287 | 281 | 287 | 364 |
|  | .5 | 234 | 199 | 185 | 199 | 234 |
| 0.75 | .2 | 645 | 509 | 487 | 509 | 645 |
|  | .3 | 288 | 227 | 222 | 227 | 288 |
|  | .4 | 163 | 129 | 126 | 129 | 163 |
|  | .5 | 105 | 83 | 82 | 83 | 105 |

*Note*. Pearson correlation (*r*), standard deviations (*SD*) and quantiles (τ).

Table S5 shows that the required sample size is lowest when only the .50 quantile is
estimated (column 5), and that it increases for higher and lower quantiles. In our study, the
average standard deviation of the 20 values at T1 is .75, with the smallest being .38 (power (dominance)). The average correlation between values at T1 and T2 was .55, with the lowest being .42 (face). At these levels (*SD* = .75, *r* = .4), the required sample size for estimation of the most extreme quantiles (τ = .2 and τ = .8) is 232.

The average standard deviation for the 15 traits at T1 is .80 with only one being less than .70 (.69). The average Pearson correlation between traits at T1 and T2 was .75, with the smallest being .66 (agreeableness (compassion)). As sample size requirements decrease with increasing *r*, the required sample size was also satisfied.

**References**

Gong, Z. (2016). *Estimation of Sample Size and Power for Quantile Regression* (Doctoral
dissertation), Queen’s University Kingston, Ontario, Canada.

**Appendix C: Reliability Testing**

**Values reliability**

We assessed the reliability of respondents’ value choices by examining the number of times each value was chosen as most important by each individual. In best-worst scaling, reliability of the data can be assessed by examining the consistency of choice. Specifically, we considered responses to be reliable when respondents chose at least one value-item as most important four or five of the five times it appeared across all sets. At Time 1, 2,633 participants did so and at Time 2 2,595 did so, yielding value estimates of 0.92 and 0.90, respectively (see Table S6).

**Table S6**

*Times a respondent’s most important value was chosen*

| Times chosen | T1 Values | | T2 Values | |
| --- | --- | --- | --- | --- |
|  | Frequency | Percentage | Frequency | Percentage |
| 0 | 1 | 0.0 | 2 | 0.1 |
| 1 | 5 | 0.2 | 6 | 0.2 |
| 2 | 42 | 1.5 | 57 | 2.0 |
| 3 | 194 | 6.7 | 215 | 7.5 |
| 4 | 981 | 34.1 | 923 | 32.1 |
| 5 | 1652 | 57.5 | 1672 | 58.2 |

*Note*. *n* = 2,875.

**Trait reliability**

**Table S7**

*Time 1 trait items rotated component matrix (acquiescence removed)*

| Item | Component | | | | |
| --- | --- | --- | --- | --- | --- |
|  | 1 | 2 | 3 | 4 | 5 |
| BFI29a_NE_EmoR | .779 | -.136 | -.100 | -.059 | -.037 |
| BFI04a_NE_AnxR | .769 | -.130 | -.099 | -.052 | -.145 |
| BFI34a_NE_Anx | .756 | .019 | -.009 | -.023 | -.170 |
| BFI54a_NE_Dep | .729 | -.136 | -.118 | -.043 | -.202 |
| BFI39a_NE_Dep | .719 | -.074 | -.069 | -.021 | -.192 |
| BFI19a_NE_Anx | .687 | .012 | -.163 | .010 | -.050 |
| BFI59a_NE_Emo | .686 | -.196 | -.122 | -.101 | .083 |
| BFI49a_NE_AnxR | .685 | .021 | .045 | .058 | -.103 |
| BFI14a_NE_Emo | .654 | -.226 | -.236 | -.058 | -.017 |
| BFI24a_NE_DepR | .649 | -.217 | -.181 | -.123 | -.222 |
| BFI44a_NE_EmoR | .624 | -.273 | -.054 | -.032 | .168 |
| BFI09a_NE_DepR | .585 | -.139 | -.278 | -.120 | -.251 |
| BFI03a_C_OrgR | -.057 | .733 | .011 | -.043 | .124 |
| BFI33a_C_Org | -.060 | .717 | .073 | -.095 | .072 |
| BFI38a_C_Pro | -.198 | .697 | .098 | .155 | .157 |
| BFI18a_C_Org | -.001 | .685 | .002 | .007 | .001 |
| BFI48a_C_OrgR | .017 | .654 | .198 | .073 | .026 |
| BFI28a_C_Res | -.103 | .615 | .171 | .068 | -.023 |
| BFI53a_C_Pro | -.167 | .611 | .291 | .179 | .047 |
| BFI08a_C_ProR | -.104 | .610 | .162 | .059 | .224 |
| BFI23a_C_ProR | -.231 | .573 | .107 | .040 | .231 |
| BFI43a_C_Res | -.188 | .528 | .412 | .150 | .006 |
| BFI13a_C_Res | -.298 | .475 | .356 | .145 | .007 |
| BFI58a_C_ResR | -.169 | .475 | .270 | .050 | -.160 |
| BFI26a_E_EneR | -.213 | .309 | .022 | .058 | .307 |
| BFI47a_A_ComR | -.008 | .163 | .694 | .108 | .128 |
| BFI02a_A_Com | .083 | .156 | .679 | .196 | .029 |
| BFI57a_A_Tru | -.227 | .053 | .647 | .049 | .178 |
| BFI27a_A_Tru | -.183 | -.013 | .644 | .019 | .080 |
| BFI52a_A_Res | -.095 | .371 | .619 | .135 | -.125 |
| BFI37a_A_ResR | -.172 | .255 | .595 | .037 | -.108 |
| BFI07a_A_Res | -.115 | .350 | .594 | .155 | -.093 |
| BFI32a_A_Com | -.094 | .283 | .587 | .133 | .001 |
| BFI12a_A_TruR | -.239 | .050 | .543 | .024 | -.015 |
| BFI22a_A_ResR | -.191 | .270 | .532 | .028 | -.223 |
| BFI42a_A_TruR | -.278 | -.112 | .418 | .028 | .214 |
| BFI17a_A_ComR | .088 | .072 | .350 | .189 | .028 |

| BFI55a_OM_IntR | -.077 | .003 | .043 | .684 | .083 |
| --- | --- | --- | --- | --- | --- |
| BFI30a_OM_CreR | -.043 | .057 | .079 | .639 | .075 |
| BFI20a_OM_Aes | .028 | -.119 | .191 | .633 | .041 |
| BFI35a_OM_Aes | -.025 | -.024 | .273 | .627 | .044 |
| BFI60a_OM_Cre | -.217 | .149 | -.043 | .599 | .188 |
| BFI45a_OM_CreR | -.089 | .147 | .139 | .587 | .092 |
| BFI10a_OM_Int | -.133 | .137 | .178 | .576 | .108 |
| BFI15a_OM_Cre | -.235 | .218 | -.013 | .571 | .147 |
| BFI25a_OM_IntR | -.078 | .097 | -.034 | .554 | .145 |
| BFI50a_OM_AesR | .057 | -.084 | .212 | .531 | .109 |
| BFI40a_OM_Int | .207 | .181 | -.044 | .526 | -.145 |
| BFI05a_OM_AesR | .073 | -.076 | .056 | .352 | .017 |
| BFI16a_E_SocR | .058 | -.065 | -.003 | .050 | .777 |
| BFI46a_E_Soc | .044 | -.026 | .107 | .087 | .701 |
| BFI31a_E_SocR | -.155 | .022 | -.001 | -.027 | .700 |
| BFI01a_E_Soc | -.266 | .029 | .270 | .056 | .678 |
| BFI06a_E_Ass | -.106 | .146 | -.213 | .210 | .588 |
| BFI21a_E_Ass | -.135 | .119 | -.288 | .242 | .566 |
| BFI56a_E_Ene | -.260 | .223 | .342 | .208 | .482 |
| BFI36a_E_AssR | -.228 | .186 | -.079 | .286 | .453 |
| BFI41a_E_Ene | -.372 | .241 | .135 | .034 | .448 |
| BFI51a_E_AssR | -.136 | .220 | -.209 | .321 | .436 |
| BFI11a_E_EneR | -.071 | .046 | .288 | .179 | .413 |

*Note*. Principal component analysis, rotation method: Varimax with Kaiser normalization, rotation converged in 7 iterations. Items ending in R reverse coded.

**Table S8**

*Time 2 trait items rotated component matrix (acquiescence removed)*

| Item | Component | | | | |
| --- | --- | --- | --- | --- | --- |
|  | 1 | 2 | 3 | 4 | 5 |
| BFI04a_NE_AnxR | .808 | -.104 | -.111 | -.057 | -.128 |
| BFI29a_NE_EmoR | .787 | -.132 | -.110 | -.043 | -.030 |
| BFI34a_NE_Anx | .775 | -.003 | -.014 | -.057 | -.166 |
| BFI54a_NE_Dep | .742 | -.161 | -.108 | -.026 | -.236 |
| BFI39a_NE_Dep | .740 | -.101 | -.088 | -.026 | -.231 |
| BFI19a_NE_Anx | .703 | .005 | -.099 | -.017 | -.062 |
| BFI49a_NE_AnxR | .693 | -.032 | .039 | .010 | -.108 |
| BFI59a_NE_Emo | .681 | -.198 | -.157 | -.109 | .060 |
| BFI14a_NE_Emo | .669 | -.177 | -.213 | -.077 | -.016 |
| BFI24a_NE_DepR | .646 | -.211 | -.177 | -.114 | -.242 |
| BFI44a_NE_EmoR | .644 | -.213 | -.090 | -.048 | .167 |
| BFI09a_NE_DepR | .630 | -.135 | -.245 | -.119 | -.238 |
| BFI03a_C_OrgR | -.078 | .754 | .026 | -.019 | .090 |
| BFI33a_C_Org | -.058 | .737 | .122 | -.070 | .048 |
| BFI38a_C_Pro | -.227 | .704 | .120 | .147 | .137 |
| BFI18a_C_Org | -.040 | .701 | .026 | .016 | -.025 |
| BFI48a_C_OrgR | .063 | .668 | .216 | .065 | -.011 |
| BFI08a_C_ProR | -.121 | .612 | .122 | .047 | .227 |
| BFI28a_C_ResR | -.143 | .587 | .242 | .077 | -.014 |
| BFI53a_C_Pro | -.147 | .586 | .315 | .172 | .079 |
| BFI23a_C_ProR | -.279 | .572 | .092 | .050 | .222 |
| BFI43a_C_Res | -.198 | .519 | .432 | .109 | .021 |
| BFI58a_C_ResR | -.165 | .447 | .324 | .037 | -.129 |
| BFI13a_C_Res | -.284 | .439 | .410 | .113 | .013 |
| BFI26a_E_EneR | -.247 | .335 | -.014 | .033 | .273 |
| BFI47a_A_ComR | .001 | .137 | .702 | .119 | .145 |
| BFI02a_A_Com | .028 | .151 | .664 | .188 | .049 |
| BFI52a_A_Res | -.079 | .332 | .659 | .101 | -.106 |
| BFI27a_A_Tru | -.195 | -.003 | .630 | .025 | .094 |
| BFI07a_A_Res | -.109 | .315 | .613 | .173 | -.062 |
| BFI37a_A_ResR | -.143 | .226 | .609 | .026 | -.094 |
| BFI57a_A_Tru | -.246 | .047 | .603 | .083 | .202 |
| BFI32a_A_Com | -.119 | .279 | .586 | .183 | -.003 |
| BFI22a_A_ResR | -.141 | .197 | .539 | .017 | -.271 |
| BFI12a_A_TruR | -.257 | .050 | .517 | .045 | -.035 |
| BFI42a_A_TruR | -.318 | -.103 | .392 | .023 | .230 |
| BFI17a_A_ComR | .075 | .043 | .374 | .153 | .048 |

| BFI30a_OM_CreR | -.061 | .065 | .115 | .678 | .080 |
| --- | --- | --- | --- | --- | --- |
| BFI55a_OM_IntR | -.114 | -.001 | .072 | .676 | .093 |
| BFI20a_OM_Aes | .029 | -.095 | .170 | .655 | .042 |
| BFI35a_OM_Aes | -.008 | -.029 | .223 | .652 | .046 |
| BFI60a_OM_Cre | -.213 | .145 | -.020 | .615 | .195 |
| BFI10a_OM_Int | -.133 | .141 | .186 | .586 | .097 |
| BFI45a_OM_CreR | -.123 | .133 | .141 | .577 | .108 |
| BFI25a_OM_IntR | -.095 | .110 | -.013 | .563 | .156 |
| BFI15a_OM_Cre | -.251 | .237 | -.006 | .554 | .105 |
| BFI50a_OM_AesR | .072 | -.100 | .225 | .538 | .104 |
| BFI40a_OM_Int | .193 | .212 | -.025 | .523 | -.100 |
| BFI05a_OM_AesR | .038 | -.094 | .033 | .402 | .010 |
| BFI16a_E_SocR | .038 | -.063 | -.015 | .025 | .789 |
| BFI31a_E_SocR | -.144 | .039 | .018 | .000 | .734 |
| BFI46a_E_Soc | -.002 | -.043 | .159 | .061 | .715 |
| BFI01a_E_Soc | -.275 | .014 | .253 | .055 | .688 |
| BFI06a_E_Ass | -.103 | .148 | -.205 | .184 | .610 |
| BFI21a_E_Ass | -.108 | .106 | -.314 | .239 | .569 |
| BFI56a_E_Ene | -.304 | .207 | .309 | .222 | .494 |
| BFI36a_E_AssR | -.185 | .198 | -.063 | .243 | .481 |
| BFI41a_E_Ene | -.397 | .244 | .069 | .076 | .425 |
| BFI11a_E_EneR | -.132 | .042 | .263 | .202 | .422 |
| BFI51a_E_AssR | -.115 | .227 | -.200 | .301 | .414 |

*Note.* Principal component analysis, rotation method: Varimax with Kaiser normalization. Rotation converged in 7 iterations. Items ending in R reverse coded.

**Table S9**

*Trait alphas*

| Trait | Facet | Items | Cronbach’s α | |
| --- | --- | --- | --- | --- |
|  |  |  | T1 | T2 |
| Extraversion |  | 12 | .844 | .849 |
|  | Sociability | 4 | .815 | .832 |
|  | Assertiveness | 4 | .777 | .769 |
|  | Energy | 4 | .715 | .730 |
| Agreeableness |  | 12 | .848 | .846 |
|  | Compassion | 4 | .662 | .663 |
|  | Respectfulness | 4 | .769 | .761 |
|  | Trust | 4 | .722 | .725 |
| Conscientiousness |  | 12 | .883 | .886 |
|  | Organization | 4 | .820 | .831 |
|  | Productiveness | 4 | .777 | .775 |
|  | Responsibility | 4 | .752 | .745 |
| Negative Emotionality |  | 12 | .919 | .927 |
|  | Anxiety | 4 | .824 | .843 |
|  | Depression | 4 | .845 | .866 |
|  | Emotional Volatility | 4 | .830 | .832 |
| Open-Mindedness |  | 12 | .831 | .841 |
|  | Intellectual Curiosity | 4 | .662 | .682 |
|  | Aesthetic Sensitivity | 4 | .722 | .732 |
|  | Creative Imagination | 4 | .755 | .766 |

**Appendix D: Quantile Correlation Tables with Confidence Estimates**

**Table S10**

*Quantile correlation results with confidence estimates: personal values*

| Value (refined) | Pearson | τ = .2 | τ = .3 | τ = .4 | τ = .5 | τ = .6 | τ = .7 | τ = .8 |
| --- | --- | --- | --- | --- | --- | --- | --- | --- |
| Universalism (animals) | **0.75** | **0.48** | **0.63** | **0.73** | **0.89** | **1.00** | **0.95** | **0.95** |
|  | 0.74 | -0.48 | -0.31 | -0.22 |  | 0.11 | 0.05 | -0.00 |
|  | 0.77 | -0.33 | -0.18 | -0.07 |  | 0.18 | 0.13 | 0.14 |
| Universalism (nature) | **0.67** | **0.41** | **0.50** | **0.61** | **0.76** | **0.86** | **0.86** | **0.90** |
|  | 0.65 | -0.46 | -0.37 | -0.25 |  | -0.01 | -0.01 | 0.03 |
|  | 0.69 | -0.28 | -0.20 | -0.09 |  | 0.14 | 0.14 | 0.19 |
| Tradition | **0.66** | **0.37** | **0.47** | **0.58** | **0.68** | **0.71** | **0.84** | **0.90** |
|  | 0.64 | -0.37 | -0.27 | -0.18 |  | -0.03 | 0.08 | 0.18 |
|  | 0.68 | -0.22 | -0.12 | -0.00 |  | 0.14 | 0.27 | 0.31 |
| Achievement | **0.61** | **0.33** | **0.43** | **0.53** | **0.62** | **0.73** | **0.87** | **0.90** |
|  | 0.59 | -0.34 | -0.25 | -0.15 |  | 0.05 | 0.18 | 0.21 |
|  | 0.63 | -0.20 | -0.09 | -0.00 |  | 0.18 | 0.31 | 0.36 |
| Universalism (concern) | **0.58** | **0.41** | **0.50** | **0.67** | **0.74** | **0.76** | **0.75** | **0.71** |
|  | 0.56 | -0.35 | -0.26 | -0.10 |  | -0.08 | -0.05 | -0.06 |
|  | 0.61 | -0.27 | -0.16 | -0.01 |  | 0.10 | 0.06 | 0.05 |
| Stimulation | **0.58** | **0.27** | **0.43** | **0.54** | **0.63** | **0.76** | **0.81** | **0.79** |
|  | 0.55 | -0.41 | -0.26 | -0.15 |  | 0.06 | 0.13 | 0.07 |
|  | 0.60 | -0.26 | -0.14 | -0.03 |  | 0.19 | 0.23 | 0.21 |
| Conformity (rules) | **0.58** | **0.34** | **0.41** | **0.50** | **0.63** | **0.76** | **0.86** | **0.80** |
|  | 0.55 | -0.35 | -0.31 | -0.17 |  | 0.05 | 0.16 | 0.12 |
|  | 0.60 | -0.20 | -0.14 | -0.04 |  | 0.22 | 0.32 | 0.31 |
| Power (resources) | **0.57** | **0.25** | **0.27** | **0.45** | **0.53** | **0.67** | **0.82** | **0.92** |
|  | 0.55 | -0.36 | -0.31 | -0.15 |  | 0.07 | 0.22 | 0.31 |
|  | 0.60 | -0.20 | -0.17 | -0.00 |  | 0.21 | 0.38 | 0.46 |
| Hedonism | **0.55** | **0.31** | **0.44** | **0.53** | **0.66** | **0.71** | **0.77** | **0.73** |
|  | 0.53 | -0.39 | -0.27 | -0.17 |  | 0.01 | 0.03 | 0.02 |
|  | 0.58 | -0.28 | -0.15 | -0.06 |  | 0.13 | 0.17 | 0.17 |
| Benevolence (dependability) | **0.53** | **0.50** | **0.67** | **0.73** | **0.63** | **0.67** | **0.63** | **0.50** |
|  | 0.51 | -0.17 | -0.03 | 0.01 |  | -0.00 | -0.09 | -0.17 |
|  | 0.56 | -0.06 | 0.12 | 0.13 |  | 0.09 | 0.05 | -0.10 |
| Self-direction (action) | **0.52** | **0.25** | **0.41** | **0.55** | **0.64** | **0.67** | **0.64** | **0.76** |
|  | 0.48 | -0.49 | -0.33 | -0.18 |  | -0.06 | -0.11 | 0.02 |
|  | 0.55 | -0.34 | -0.18 | -0.05 |  | 0.10 | 0.06 | 0.15 |
| Universalism (tolerance) | **0.52** | **0.41** | **0.51** | **0.60** | **0.64** | **0.66** | **0.67** | **0.58** |
|  | 0.49 | -0.30 | -0.21 | -0.11 |  | -0.08 | -0.06 | -0.12 |
|  | 0.54 | -0.18 | -0.08 | 0.02 |  | 0.07 | 0.08 | 0.04 |
| Benevolence (caring) | **0.51** | **0.50** | **0.62** | **0.58** | **0.59** | **0.50** | **0.50** | **0.50** |
|  | 0.48 | -0.13 | -0.06 | -0.07 |  | -0.13 | -0.13 | -0.13 |
|  | 0.53 | -0.02 | 0.12 | 0.11 |  | 0.06 | 0.01 | -0.02 |
| Humility | **0.51** | **0.21** | **0.31** | **0.38** | **0.48** | **0.55** | **0.71** | **0.77** |
|  | 0.48 | -0.30 | -0.24 | -0.17 |  | 0.04 | 0.17 | 0.24 |
|  | 0.54 | -0.17 | -0.11 | -0.03 |  | 0.18 | 0.30 | 0.37 |
| Self-direction (thought) | **0.49** | **0.41** | **0.47** | **0.47** | **0.53** | **0.48** | **0.56** | **0.62** |
|  | 0.46 | -0.18 | -0.13 | -0.13 |  | -0.09 | -0.02 | 0.04 |
|  | 0.52 | -0.04 | 0.02 | 0.02 |  | 0.08 | 0.13 | 0.18 |
| Conformity (interpersonal) | **0.48** | **0.29** | **0.32** | **0.38** | **0.47** | **0.50** | **0.63** | **0.76** |
|  | 0.46 | -0.25 | -0.21 | -0.15 |  | -0.02 | 0.06 | 0.20 |
|  | 0.51 | -0.14 | -0.07 | -0.00 |  | 0.15 | 0.23 | 0.33 |
| Security (societal) | **0.47** | **0.45** | **0.54** | **0.58** | **0.50** | **0.55** | **0.50** | **0.50** |
|  | 0.44 | -0.08 | -0.04 | -0.00 |  | -0.01 | -0.03 | -0.10 |
|  | 0.50 | -0.00 | 0.11 | 0.08 |  | 0.11 | 0.12 | 0.05 |
| Security (personal) | **0.46** | **0.29** | **0.50** | **0.61** | **0.50** | **0.50** | **0.67** | **0.58** |
|  | 0.43 | -0.38 | -0.17 | -0.07 |  | -0.17 | -0.02 | -0.10 |
|  | 0.49 | -0.17 | 0.00 | 0.17 |  | 0.03 | 0.17 | 0.19 |
| Power (dominance) | **0.46** | **0.31** | **0.25** | **0.35** | **0.40** | **0.52** | **0.63** | **0.75** |
|  | 0.43 | -0.21 | -0.23 | -0.12 |  | 0.05 | 0.12 | 0.21 |
|  | 0.49 | -0.04 | -0.08 | 0.04 |  | 0.19 | 0.30 | 0.46 |
| Face | **0.42** | **0.27** | **0.29** | **0.33** | **0.42** | **0.50** | **0.55** | **0.69** |
|  | 0.39 | -0.24 | -0.24 | -0.19 |  | -0.02 | 0.04 | 0.18 |
|  | 0.45 | -0.08 | -0.06 | -0.01 |  | 0.16 | 0.26 | 0.35 |

*Note*. *n* = 2875. The first row for each value (ordered by Pearson correlation strength) presents the estimated correlations for value-stability over a 2-year period (in bold). The second and third rows present estimated 95% confidence intervals for the Pearson correlation and the bootstrapped 95% confidence intervals for the difference between the median (τ =.5) quantile correlation and each of the quantile correlations. If the quantile correlation confidence intervals do not include zero, the quantile correlation differs significantly from the median correlation. Quantile correlations highlighted in green are significantly stronger and those in yellow are significantly weaker than the median correlation.

**Table S11**

*Quantile correlation results with confidence estimates: personality traits*

| Trait (Facet) | Pearson corr. | τ = .2 | τ = .3 | τ = .4 | τ = .5 | τ = .6 | τ = .7 | τ = .8 |
| --- | --- | --- | --- | --- | --- | --- | --- | --- |
| Extraversion (Sociability) | **0.83** | **0.83** | **0.85** | **0.87** | **0.93** | **0.91** | **0.91** | **0.85** |
|  | 0.81 | -0.13 | -0.10 | -0.08 |  | -0.07 | -0.07 | -0.10 |
|  | 0.84 | -0.08 | -0.01 | -0.03 |  | 0.01 | 0.01 | -0.06 |
| Conscientiousness (Organization) | **0.78** | **0.88** | **0.90** | **0.88** | **0.91** | **0.85** | **0.75** | **0.65** |
|  | 0.77 | -0.10 | -0.07 | -0.09 |  | -0.12 | -0.21 | -0.32 |
|  | 0.8 | 0.01 | 0.06 | 0.04 |  | -0.01 | -0.11 | -0.21 |
| Negative Emotionality (Anxiety) | **0.78** | **0.80** | **0.82** | **0.84** | **0.89** | **0.84** | **0.82** | **0.77** |
|  | 0.77 | -0.12 | -0.10 | -0.08 |  | -0.09 | -0.10 | -0.15 |
|  | 0.8 | -0.01 | -0.00 | 0.02 |  | 0.02 | -0.00 | -0.05 |
| Negative Emotionality (Depression) | **0.78** | **0.75** | **0.80** | **0.83** | **0.85** | **0.85** | **0.87** | **0.84** |
|  | 0.77 | -0.14 | -0.09 | -0.06 |  | -0.04 | -0.02 | -0.04 |
|  | 0.80 | -0.07 | -0.02 | 0.01 |  | 0.03 | 0.07 | 0.03 |
| Extraversion (Assertiveness) | **0.78** | **0.80** | **0.82** | **0.83** | **0.84** | **0.84** | **0.79** | **0.78** |
|  | 0.76 | -0.08 | -0.06 | -0.05 |  | -0.04 | -0.08 | -0.10 |
|  | 0.79 | -0.01 | 0.01 | 0.02 |  | 0.03 | -0.00 | -0.01 |
| Open-Mindedness (Aesthetic Sensitivity) | **0.77** | **0.82** | **0.83** | **0.82** | **0.82** | **0.80** | **0.79** | **0.74** |
|  | 0.76 | -0.08 | -0.07 | -0.08 |  | -0.10 | -0.11 | -0.15 |
|  | 0.79 | 0.03 | 0.04 | 0.03 |  | 0.01 | 0.00 | -0.05 |
| Conscientiousness (Productiveness) | **0.76** | **0.80** | **0.79** | **0.85** | **0.85** | **0.85** | **0.80** | **0.71** |
|  | 0.74 | -0.16 | -0.16 | -0.10 |  | -0.10 | -0.15 | -0.24 |
|  | 0.78 | 0.00 | -0.00 | 0.04 |  | 0.04 | -0.01 | -0.09 |
| Negative Emotionality (Emotional Volatility) | **0.76** | **0.72** | **0.80** | **0.81** | **0.83** | **0.83** | **0.80** | **0.81** |
|  | 0.74 | -0.14 | -0.07 | -0.07 |  | -0.04 | -0.06 | -0.07 |
|  | 0.77 | -0.08 | -0.00 | 0.01 |  | 0.03 | 0.00 | -0.00 |
| Agreeableness (Trust) | **0.74** | **0.76** | **0.77** | **0.77** | **0.80** | **0.76** | **0.75** | **0.75** |
|  | 0.72 | -0.06 | -0.06 | -0.06 |  | -0.06 | -0.07 | -0.10 |
|  | 0.76 | 0.03 | 0.04 | 0.03 |  | 0.03 | 0.02 | 0.00 |
| Open-Mindedness (Intellectual Curiosity) | **0.73** | **0.76** | **0.76** | **0.77** | **0.79** | **0.77** | **0.77** | **0.73** |
|  | 0.72 | -0.12 | -0.11 | -0.10 |  | -0.10 | -0.10 | -0.14 |
|  | 0.75 | 0.01 | 0.02 | 0.03 |  | 0.02 | 0.02 | -0.02 |
| Extraversion  (Energy Level) | **0.73** | **0.76** | **0.77** | **0.77** | **0.78** | **0.79** | **0.76** | **0.75** |
|  | 0.72 | -0.07 | -0.06 | -0.05 |  | -0.05 | -0.07 | -0.09 |
|  | 0.75 | 0.01 | 0.02 | 0.03 |  | 0.03 | 0.01 | -0.01 |
| Open-Mindedness (Creative Imagination) | **0.73** | **0.77** | **0.79** | **0.77** | **0.77** | **0.77** | **0.77** | **0.71** |
|  | 0.72 | -0.05 | -0.04 | -0.05 |  | -0.05 | -0.05 | -0.11 |
|  | 0.75 | 0.05 | 0.03 | 0.04 |  | 0.04 | 0.03 | -0.05 |
| Agreeableness (Respectfulness) | **0.72** | **0.77** | **0.80** | **0.82** | **0.89** | **0.79** | **0.73** | **0.60** |
|  | 0.70 | -0.16 | -0.13 | -0.11 |  | -0.14 | -0.20 | -0.34 |
|  | 0.74 | -0.02 | 0.01 | 0.02 |  | -0.00 | -0.07 | -0.19 |

| Conscientiousness (Responsibility) | **0.71** | **0.75** | **0.75** | **0.82** | **0.76** | **0.82** | **0.77** | **0.67** |
| --- | --- | --- | --- | --- | --- | --- | --- | --- |
|  | 0.69 | -0.08 | -0.05 | -0.00 |  | -0.00 | -0.03 | -0.14 |
|  | 0.73 | 0.04 | 0.01 | 0.07 |  | 0.07 | 0.04 | -0.08 |
| Agreeableness (Compassion) | **0.66** | **0.64** | **0.75** | **0.77** | **0.75** | **0.80** | **0.73** | **0.60** |
|  | 0.63 | -0.17 | -0.07 | -0.04 |  | -0.01 | -0.08 | -0.20 |
|  | 0.68 | -0.05 | 0.00 | 0.07 |  | 0.07 | 0.00 | -0.13 |

*Note.* *n* = 2424. The first row for each trait (ordered by Pearson correlation strength) presents the estimated correlations for trait-stability over a 2-year period (in bold). The second and third rows present estimated 95% confidence intervals for the Pearson correlation and the bootstrapped 95% confidence intervals for the difference between the median (τ = .5) quantile correlation and each of the quantile correlations. If the quantile correlation confidence intervals do not include zero, the quantile correlation differs significantly from the median correlation. Quantile correlations highlighted in green are significantly stronger and those in yellow are significantly weaker than the median correlation.

**Appendix F: Comparing Best-Worst and Rating Scale Measures of Values**

To examine whether patterns observed in quantile correlations results are robust across different types of measures, values data was collected using both rating scales and best-worst scaling within the same sample. Specifically, we tested whether (a) neighboring values in the theoretical values structure correlate more strongly at higher levels of value importance than at lower levels, and (b) whether data collected with different measures would correlate more strongly at higher levels of values importance than at lower levels. In this case, the two most highly correlated values were examined. Finding support for these propositions would add confidence that our results were not due to measurement differences between values and traits.

**Method**

An online sample of adults from the USA, UK, Australia and Canada completed both the Schwartz Values Best Worst Scale (SVBWS; Lee et al., 2008) and a modified version of the Portrait Values Questionnaire (PVQ; Schwartz et al, 2001), as part of a larger study conducted in 2023. Data collection for this study was approved by [University withheld for blind review] Human Ethics Committee (2022/ET000746).

***Participants and Procedure***

An online panel survey was fielded by a full-service market research agency, with a target of 2,000 completed surveys in the USA and in the UK, and 1,000 completed surveys in Australia and in Canada. Quotas were used to obtain nationally representative samples on the basis of age, gender, ethnicity, region, and income. In total, the sample comprised 5,917 adults (1,952 USA; 1,976 UK; 1,007 Australian; 982 Canadian). The mean age was 47.8 years (*SD* = 17.5; min = 18, max = 100), with 53% being women, 42% reporting their highest level of education to be secondary school (i.e., high school) and 52% reporting post-secondary education.

All respondents first completed the SVBWS (Lee et al., 2008) and then completed a modified version of the PVQ (Schwartz et al., 2001). The modified PVQ included 22 items: 21 from the Human Values Scale of the European Social Survey [ESS21] and an additional universalism (nature) item from the PVQ-40 (see Schwartz, 2021) to provide two items for each of the 11 values being measured. In addition, a mid-point between each of the six labeled scale points (*very much like me, like me, somewhat like me, a little like me, not like me, and not like me at all*) was added to increase variability. For the SVBWS, scores were obtained for 11 values, using the square root ratio scoring method, following Lee and colleagues (2008). For the PVQ, the same values were scored by first ipsatizing the 22 items (i.e., taking the mean of all value-item scores from each value-item score), as suggested by Schwartz (1992), and then averaging the two relevant items for each of the 11 values.

Quantile correlations were estimated at seven points along the distribution of value importance (representing the 20^th^, 30^th^, 40^th^, … to 80^th^ percentiles), in addition to Pearson correlations, following the analysis in the main paper.

All data and syntax are available at: <https://osf.io/s2c74/?view_only=39039a43acf248ee81b9f6145bad9b60>.

**Results**

Universalism (society) and universalism (nature) values were chosen for analysis as they were comparatively highly correlated with each other both in the SVBWS and PVQ data, and correlated at the same level (*r* = .20) in both measures. Values descriptive statistics are shown in Table S12. Universalism (society) means were higher than universalism (nature) means, with similar standard deviations for both measure types.

**Table S12**

*Scale comparison: values descriptive statistics*

| Variable | Mean | *SD* | Min. | Max. |
| --- | --- | --- | --- | --- |
| Universalism (societal): SBWVS | 1.52 | 0.89 | 0.25 | 4.00 |
| Universalism (nature): SBWVS | 1.46 | 0.83 | 0.25 | 4.00 |
| Universalism (societal): PVQ | 0.56 | 0.87 | -3.00 | 3.73 |
| Universalism (nature): PVQ | 0.25 | 0.94 | -3.55 | 3.23 |

*Note*. *n* = 5,917.

Quantile correlation estimates were first examined between the two neighboring values using data from the same scale, and then between the same values using data from different scales. Figure S1 depicts the results of these analyses. Row 1 shows that correlations between the same value measured with different scales were stronger at higher levels of value importance and weaker at lower levels, as expected. Row 2 shows that the correlations between neighboring values measured with both the SVBWS and PVQ were stronger at higher levels of value importance.

In the main paper, jittering data (as employed in Lee et al., 2022) to increase the continuity of best-worst values scores was not necessary. However, in Figure S1, row 2, column 1, some failure of estimation at the lower end of the SVBWS distribution was evident, due to a lack of continuity at these points, and the relatively low correlation between the pair of variables. Consequently, the analyses were repeated with jittered data for SVBWS scores (following Machado & Silva, 2005), as a point of comparison. The “jitters” had a mean of zero and a standard deviation of .2 times the minimum distance between unique values scores for that value item, leaving the jittered scores equivalent, on average, to raw scores. Figure S2 depicts the same analyses as above using the jittered SVBWS data. Results are broadly equivalent to those from the prior analyses, albeit with slightly wider confidence intervals, however we can observe that in Figure S2, row 2, column 2, jittering was effective in addressing estimation failure.

In sum, these supplementary results indicate that there is greater stability across different scale types at higher levels of value importance than at lower levels, and further, that relations between neighboring values are stronger at higher levels of value importance than at lower levels for both best-worst scaling and rating scales. This suggests that the phenomenon observed in the main paper is unlikely to be due entirely to scale dependence; however, there are differences with the scales used in this analysis and those in the main paper. Thus, further research is needed to replicate these findings, and extend them to both trait measures and the temporal stability of values and traits.

**Figure S1**

*Quantile correlation results: universalism values (within and between alternative measures)*

| Universalism (nature):  SVBWS & PVQ | Universalism (society):  SVBWS & PVQ |
| --- | --- |
| 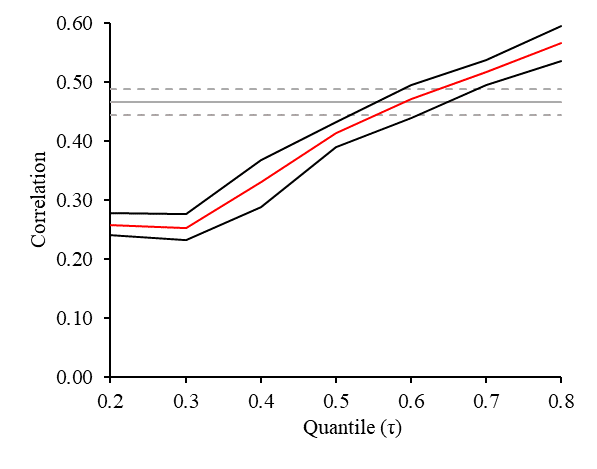 | 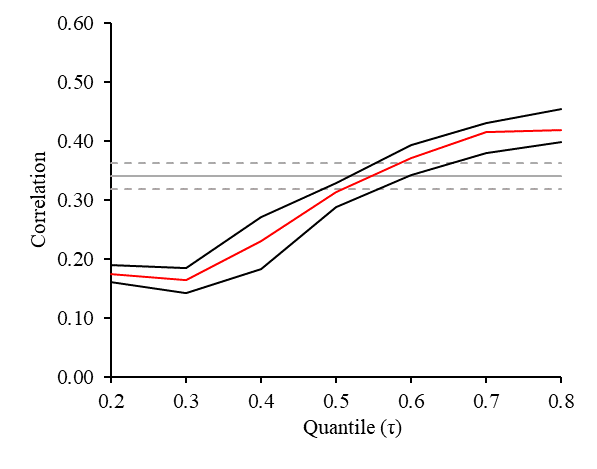 |
| SVBWS:  Universalism (nature) and (society) | PVQ:  Universalism (nature) & (society) |
| 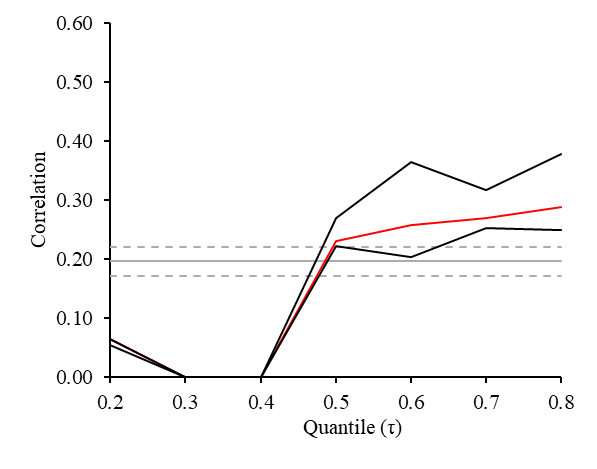 | 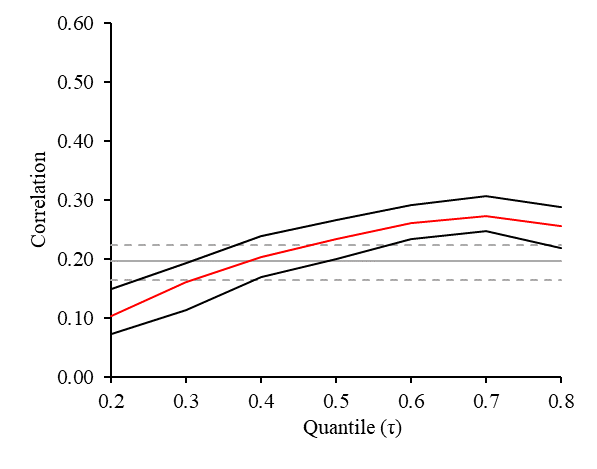 |

*Note*. *n* = 5,217. Grey lines represent the Pearson correlation, with dotted lines at 95% confidence intervals; red lines represent quantile correlations, with black lines at 95% confidence intervals.

**Figure S2**

*Quantile correlation results: universalism values (SVBWS values jittered)*

| Universalism (nature):  SVBWS & PVQ | Universalism (society):  SVBWS & PVQ |
| --- | --- |
| 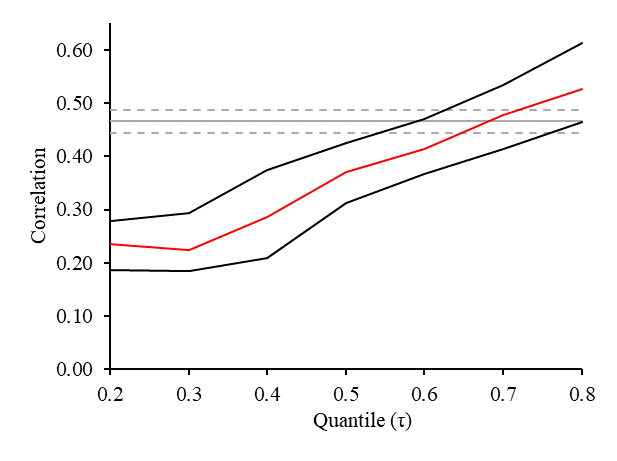 | 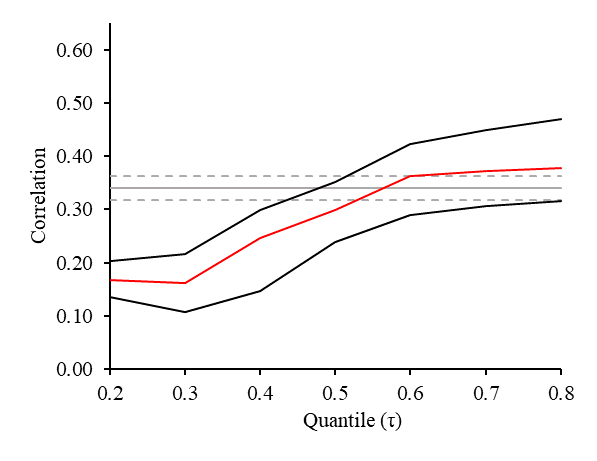 |
| SVBWS:  Universalism (nature) and (society) | PVQ:  Universalism (nature) & (society) |
| 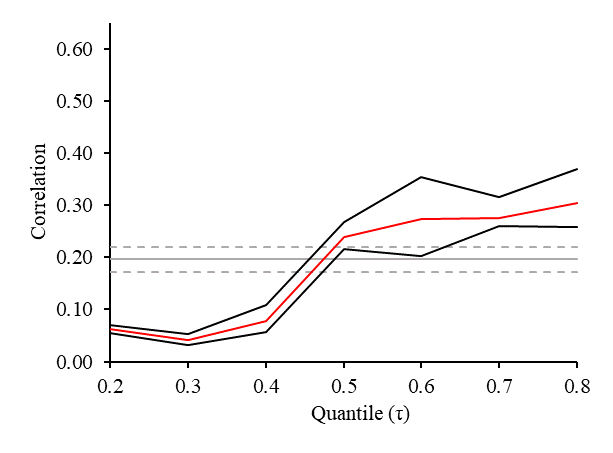 | 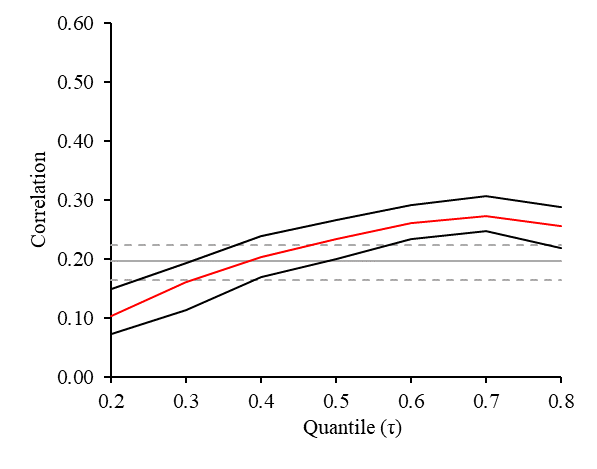 |

*Note*. *n* = 5,217. Grey lines represent the Pearson correlation, with dotted lines at 95% confidence intervals; red lines represent quantile correlations, with black lines at 95% confidence intervals.

**References**

Lee, J. A., Bardi, A., Gerrans, P., Sneddon, J., van Herk, H., Evers, U., & Schwartz, S. (2022). Are value-behavior relations stronger than previously thought? It depends on value importance. *European Journal of Personality, 36*(2), 133-148. [doi:10.1177/08902070211002965](https://doi.org/10.1177/08902070211002965).

Lee, J, Sneddon, J. N., Daly, T. M., Schwartz, S. H., Soutar, G.N. & Louviere, J. (2019). Testing and extending Schwartz refined value theory using a best-worst scaling approach. *Assessment*, *26*(2), 166-180. [doi:10.1177/1073191116683799](https://doi.org/10.1177/1073191116683799).

Lee, J. A., Soutar, G., & Louviere, J. (2008). The best–worst scaling approach: An alternative to Schwartz's values survey. *Journal of personality assessment, 90*(4), 335-347. [doi:10.1080/00223890802107925](https://doi.org/10.1080/00223890802107925).

Machado, J. A. F., & Silva, J. S. (2005). Quantiles for counts. *Journal of the American Statistical Association, 100*(472), 1226-1237. [doi:10.1198/016214505000000330](https://doi.org/10.1198/016214505000000330).

Schwartz, S. H. (1992). Universals in the content and structure of values: Theoretical advances and empirical tests in 20 countries. In M. P. Zanna (Ed.), *Advances in experimental social psychology*, Vol. 25 (pp. 1–65). Academic Press. [doi:10.1016/S0065-2601(08)60281-6](https://doi.org/10.1016/S0065-2601(08)60281-6).

Schwartz, S. H. (2021). A Repository of Schwartz Value Scales with Instructions and an Introduction. Online Readings in Psychology and Culture, 2(2). [doi:10.9707/2307-0919.1173](https://doi.org/10.9707/2307-0919.1173).

Schwartz, S. H., Melech, G., Lehmann, A., Burgess, S., Harris, M., & Owens, V. (2001). Extending the Cross-Cultural Validity of the Theory of Basic Human Values with a Different Method of Measurement. *Journal of Cross-Cultural Psychology*, *32*(5), 519-542. [doi:10.1177/0022022101032005001](https://doi.org/10.1177/0022022101032005001).
